# Pathogenic bacteria experience pervasive RNA polymerase backtracking during infection

**DOI:** 10.1128/mbio.02737-23

**Published:** 2023-12-14

**Authors:** Kaitlyn R. Browning, Houra Merrikh

**Affiliations:** 1 Department of Biochemistry, Vanderbilt University School of Medicine, Nashville, Tennessee, USA; Fred Hutchinson Cancer Center, Seattle, Washington, USA; University of Colorado Anschutz Medical Campus School of Medicine, Aurora, Colorado, USA

**Keywords:** RNAP backtracking, host-pathogen interactions, PIC-seq, *Salmonella enterica*

## Abstract

Pathogenic bacteria and their eukaryotic hosts are in a constant arms race. Hosts have numerous defense mechanisms, including some that cause DNA damage. How these host defense mechanisms impact molecular transactions along the bacterial chromosome during infection is unclear. This is partially due to the lack of a method that can detect these events in pathogens while they are within host cells. We developed and optimized a system capable of mapping and measuring levels of bacterial proteins associated with the chromosome while they are actively infecting the host (referred to as PIC-seq). Here, we focused on the dynamics of RNA polymerase (RNAP) movement and association with the chromosome in the pathogenic bacterium *Salmonella enterica* as a model system during infection. Using PIC-seq, we found that RNAP association patterns with the chromosome change during infection genome wide, including at regions that encode for key virulence genes. Importantly, we found that infection of a host significantly increases RNAP backtracking on the bacterial chromosome. RNAP backtracking is the most common form of disruption to RNAP progress on the chromosome. Interestingly, we found that genes experiencing backtracking are downregulated. We observed that the resolution of backtracked RNAPs *via* the anti-backtracking factors GreA and GreB is critical for pathogenesis and for regulation of gene expression during infection. Altogether, our findings have important implications for both efficient transcription and stalled RNAP-driven mutagenesis, which can promote antimicrobial resistance and/or hypervirulence. These results suggest that the host can accelerate the evolution of bacterial pathogens, potentially at their own expense.

**IMPORTANCE:**

Eukaryotic hosts have defense mechanisms that may disrupt molecular transactions along the pathogen’s chromosome through excessive DNA damage. Given that DNA damage stalls RNA polymerase (RNAP) thereby increasing mutagenesis, investigating how host defense mechanisms impact the movement of the transcription machinery on the pathogen chromosome is crucial. Using a new methodology we developed, we elucidated the dynamics of RNAP movement and association with the chromosome in the pathogenic bacterium *Salmonella enterica* during infection. We found that dynamics of RNAP movement on the chromosome change significantly during infection genome-wide, including at regions that encode for key virulence genes. In particular, we found that there is pervasive RNAP backtracking on the bacterial chromosome during infections and that anti-backtracking factors are critical for pathogenesis. Altogether, our results suggest that, interestingly, the host environment can promote the development of antimicrobial resistance and hypervirulence as stalled RNAPs can accelerate evolution through increased mutagenesis.

## INTRODUCTION

Antimicrobial resistance is an urgent threat to human health. Bacterial pathogens continue to evolve at a rate that outpaces almost all old and new therapeutics. Resolving this problem requires a general understanding of how hosts and pathogens interact during infection. Numerous studies over the past few decades have demonstrated how different pathogens reach, invade, and proliferate within their target hosts. However, how the host impacts the pathogen during infection at the molecular level, specifically the complexes that function on the bacterial chromosome, has not been thoroughly investigated.

Bacterial pathogens face significant challenges from the moment of initial contact with mammalian hosts. They must successfully survive harsh environments and regulate the expression of key virulence factors like pathogenicity island-encoded secretion systems, flagella, ion transporters, and stress response genes. For enteric intracellular pathogens, ingested cells must respond to the acidic pH (1) and the presence of reactive nitrogen species (2) within the host stomach. They must also overcome nutritional barriers caused by dense microbiota layers (3) and withstand antimicrobial peptides released by host cells (4). The bacteria are also heavily bombarded by oxidative stress (5–9). Although pathogenic bacteria are generally well equipped to respond to these host defense mechanisms, any number of these stresses during infection could lead to an accumulation of DNA damage, especially oxidative stress. Indeed, studies in *Salmonella enterica* (10, 11), *Staphylococcus aureus* (12)*,* and *Helicobacter pylori* (13) demonstrate the critical necessity of DNA repair during infection.

DNA damage could have numerous consequences for bacterial cells during infection. It is well documented that obstacles on the DNA template, such as damaged template bases or DNA-protein adducts, stall transcription elongation complexes (14–17). Backtracking is the most common type of RNA polymerase (RNAP) stalling (18). When backtracked, the active site of RNAP becomes disengaged, the polymerase physically moves backward along the DNA, and the 3′ end of the nascent RNA is extruded through the secondary channel of the polymerase (19–21). The RNAP remains in this stable state until its catalytic activity is restored by anti-backtracking factors.

Backtracking could have a profound impact on RNAP dynamics and transcription genome wide, with far-reaching consequences. For instance, the movement of the replication forks may be perturbed within chromosomal regions packed with stalled RNAPs. Disruptions to RNAP progression could also increase mutagenesis by way of inducing transcription-coupled repair, which was recently shown to be mutagenic (14, 22–26). In this way, it is possible that RNAP backtracking is increased within the pathogen during infection, thereby increasing the chances of generating hypervirulent strains and facilitating AMR development.

Beyond these possibilities, any impediment to RNAP movement induced by the host is likely to significantly abrogate the transcriptional profile of the pathogen during infection. Gene expression programs that respond to the host environment are highly intricate and tightly regulated (27, 28). Thus, any unresolved disruptions to RNAP progression during infection could be catastrophic to the survival of the pathogen. In *S. enterica* grown in broth culture, the activity of anti-backtracking factors GreA and GreB in regulating transcriptional pauses is critical for the expression of metabolic outputs that promote cellular survival under oxidative stress (29). Interestingly, both factors have also been demonstrated to be important for the expression of genes within *Salmonella* pathogenicity islands (SPIs) 1 and 2, even outside the host environment (29, 30). However, how a host impacts RNAP dynamics along the pathogen chromosome specifically during infection, and any consequences thereof, has not been investigated. The pervasiveness of RNAP backtracking along the pathogenic chromosome, whether the host environment affects these levels, and if this yields Gre factors critical for virulence gene expression during infection remain open questions. This is partially due to the lack of a precise method capable of detecting changes at the molecular level on the chromosome of pathogens while they are within host cells.

Here, we investigated how the host impacts RNAP movement on the chromosome of a bacterial pathogen, focusing our investigation on RNAP backtracking. We used the facultative intracellular pathogen *Salmonella enterica* serovar Typhimurium (*S*. Typhimurium) as our model organism. We developed a modified chromatin immunoprecipitation (ChIP) method, post-infection ChIP followed by deep sequencing (PIC-seq), that we used to measure RNAP density and changes in RNAP movement on the bacterial chromosome while the pathogen is inside the host cell. Our data strongly suggest that RNAP backtracking is significantly more prevalent during infection compared to cells grown in broth culture. Furthermore, we find that these disruptions to RNAP progression occur across the genome, including at regions encoding for key *S*. Typhimurium virulence genes. Our results suggest that Gre factors regulate gene expression during infection. We also show that the resolution of backtracking is critical to pathogenesis, highlighting a key role for anti-backtracking factors GreA and GreB in infections. Our results suggest that RNAP movement is substantially impacted by the host genome wide and that the resolution of these disruptions is critical for the survival of pathogens during infection.

## RESULTS

### The development of a method to measure changes in bacterial protein association with the chromosome during infection

**Fig 1:**
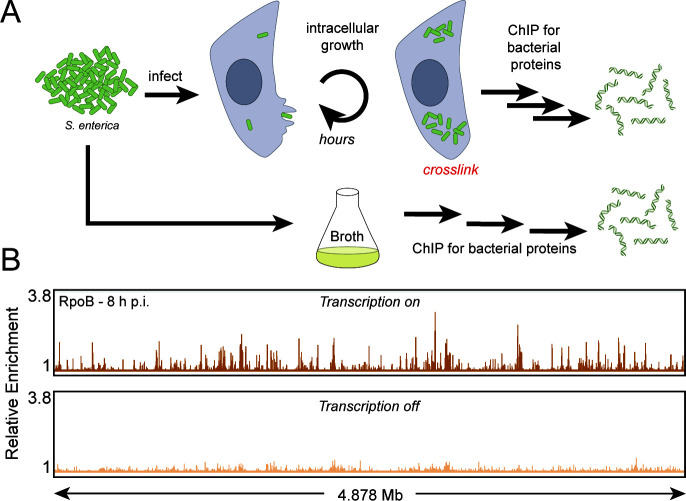
PIC-seq method can be used to measure RNAP occupancy during infection. (**A**) Schematic of PIC-seq in bacteria cells from infected HeLa. Eukaryotic host cells are infected with pathogenic bacteria cells. Once the infection has progressed for a given time, the system is chemically fixed to lock protein-chromosome interactions in place in time. Subsequent immunoprecipitation can be performed. (**B**) Representative example of *S*. Typhimurium RNAP occupancy 8 h post-infection (p.i.) of HeLa cells as determined by PIC-seq of RpoB (the beta subunit of RNAP). Inhibiting transcription with rifampicin during the infection (“transcription off”) abrogates RpoB (RNAP) enrichment genome wide. Relative enrichment is defined as the ratio of IP and input read counts normalized to total read depth. The total genome size is 4.878 Mb.

We hypothesized that RNAP backtracking is more prevalent during infection genome wide and that resolution is critical to pathogenesis. To test this hypothesis, we needed a system that would allow us to map RNAP occupancy on the chromosome of the pathogen specifically while the bacteria still resided within host cells. A successful method would allow the isolation of protein-chromatin interactions without any signal loss or creation of artifacts during sample processing. To our knowledge, such a method did not exist. We developed and optimized a system whereby well-established *ex vivo* infection protocols are paired with ChIP of bacterial proteins, followed by deep sequencing of the immunoprecipitating DNA (Fig. 1A). By chemically fixing the protein-chromatin interactions while the bacterial pathogen still resides within the host cell, our method ensures that no fleeting molecular interactions are lost during sample processing and minimizes generation of artifacts, as could be the case if we first isolated the bacteria away from the host cells before fixation. PIC-seq enabled us to determine RNAP dynamics at the molecular level specifically during infection.

We used *S*. Typhimurium to infect HeLa cells based on highly standardized protocols (9, 31–33). At defined timepoints post-infection, bacterial proteins were chemically crosslinked to the DNA while the bacteria still resided within the host cell. Both bacterial and host cells were then lysed and their DNA was fragmented by sonication. RpoB, the β subunit of RNAP, was immunoprecipitated from the mixed lysate using a native antibody. Using Western blot analyses, we determined that the antibody is highly specific to RpoB and does not cross-react with proteins specific to HeLa cells (Fig. S1). The associated DNA was then extracted and deep sequenced. Any contaminating DNA from the host is naturally excluded from further analysis, as it does not map to the *S*. Typhimurium genome. On average, 12.4% of total reads mapped to the *S*. Typhimurium genome in our experiments, providing an average of approximately 80× coverage (approximately 2.6 million aligned reads per sample). Though we used this system to immunoprecipitate RNAP from *Salmonella*, PIC-seq can, in principle, be adapted to examine any protein of interest with a specific antibody in any host-pathogen combination.

We measured RNAP association with the pathogen genome using PIC-seq. In parallel, to determine whether our signal was specific to the bacterial pathogen, we briefly treated cells with the antibiotic rifampicin during the infection. Rifampicin inhibits bacterial transcription initiation while allowing elongating RNAPs to complete transcription, thereby reducing the RNAP signal that can be detected in ChIP experiments (34, 35). As previously shown in culture (34, 35), and expected here in the context of infection, our analysis revealed that RNAP occupancy signal was significantly reduced genome wide upon rifampicin treatment (Fig. 1B). These findings demonstrate the specificity of PIC-seq for detection of bacterial protein-chromosome interactions. To rule out the possibility that this decrease in RNAP occupancy was caused by unintentional rifampicin-induced cell death, we treated cells with carbenicillin, a different antibiotic that inhibits cell wall synthesis rather than transcription, and measured RNAP occupancy using qPCR. Treatment with carbenicillin led to significant cell death during infection (Fig. S2A). Despite this pronounced effect, carbenicillin treatment did not abrogate RNAP occupancy (Fig. S2B). This indicates that the decrease in RNAP occupancy during treatment with rifampicin is due to the inhibition of transcription, and not due to cell death, further supporting the conclusion that PIC-seq can specifically isolate bacterial pathogen chromatin-protein complexes from an infection model.

### RNAP association with virulence genes can be specifically detected during infection

**Fig 2:**
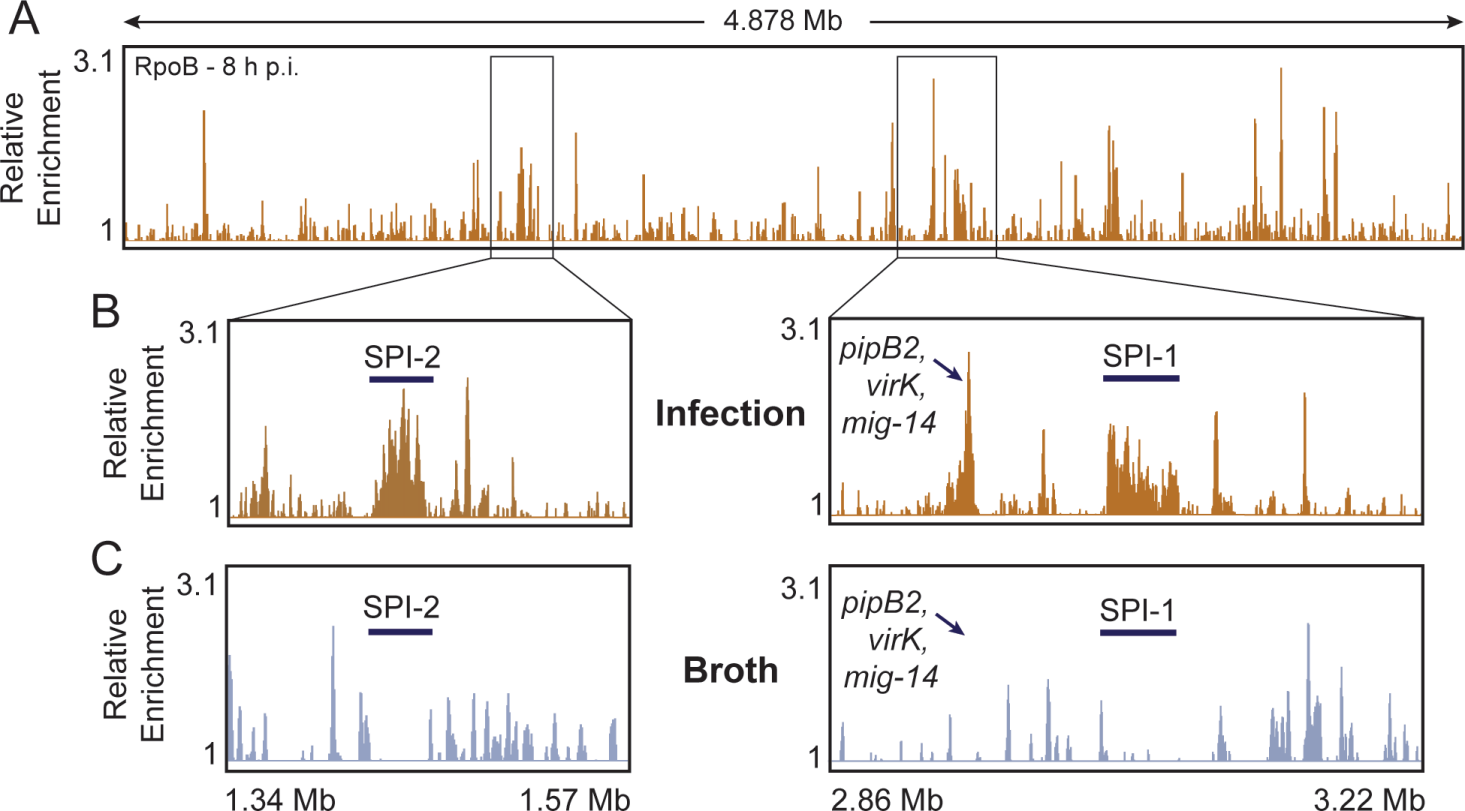
Specific detection of RNAP occupancy at *S*. Typhimurium virulence genes. (**A**) Representative example of *S*. Typhimurium RNAP occupancy 8 h post-infection (h p.i.) of HeLa cells as determined by PIC-seq of RpoB. (**B**) Reads mapped to the *S*. Typhimurium genome display areas of high RNAP enrichment during infection, notably within *Salmonella* pathogenicity islands (SPIs) and at a trio of virulence genes (*pipB2*, *virK*, and *mig-14*). (**C**) No significant RNAP signal was found at these genes in cells grown in broth culture. Relative enrichment is defined as the ratio of IP and input read counts normalized to total read depth.

To further confirm that our method can differentiate between cells grown in broth culture versus infection, we analyzed the levels of RNAP occupancy with regions encoding virulence genes (Fig. 2A; Fig. S3A). As expected, RNAP was significantly enriched at regions of the chromosome containing multiple, successive virulence genes (*Salmonella* pathogenicity islands, “SPIs”) during infection (Fig. 2B). Importantly, no significant RNAP signal was found at these genes in cells grown in broth (Fig. 2C). Similar trends were observed at a trio of virulence genes, *pipB2*, *virK*, and *mig-14*, where there is no RNAP enrichment in cells grown in broth, but clear enrichment during infection. Unsurprisingly, almost all genes with significant RNAP enrichment during infection (but not in broth culture) have a role (confirmed or putative) in virulence (Table S1). These results are consistent with previous RNA-seq data sets of *S*. Typhimurium during infection (9, 36, 37), as well as the well-defined roles of SPI-1 and SPI-2 proteins in facilitating host cell invasion and promoting intracellular survival, respectively (38). By contrast, we observed similar levels of RNAP at housekeeping gene regions, such as at ribosomal genes (rDNA), in both conditions (Fig. S3B; Table S2). Our results demonstrate that PIC-seq can specifically map and measure the levels of infection-specific RNAP association with the bacterial genome. Furthermore, the high levels of RNAP occupancy at virulence genes that we observed support prior work and the conclusion that these genes are vital during infection.

### RNAP backtracking increases significantly during infection

RNAPs naturally backtrack, especially in the face of DNA damage or other obstacles in the way. We hypothesized that RNAP backtracking is more prevalent during infection genome wide because several host defense mechanisms can damage DNA (such as through oxidative stress), potentially inhibiting the movement of the transcription machinery along the genome. In *S*. Typhimurium, the anti-backtracking factors GreA and GreB rescue backtracked RNAPs by stimulating the intrinsic endonucleolytic activity of RNAP to restore the active site (39, 40).

To test our hypothesis, we constructed an *S*. Typhimurium strain lacking GreA and GreB (41). If our hypothesis is correct, then cells lacking both Gre factors would have increased levels of stalled RNAPs during infection compared to growth in broth culture. To test this model, we measured the differences in RNAP occupancy levels across the genome in the presence (wild type, WT) and absence (Δ*greA* Δ*greB*) of these anti-backtracking factors. Since both Gre factors are directly and specifically involved in the resolution of backtracked RNAPs (39, 40), we deduced that sites where RNAP occupancy changes in the absence of Gre factors are regions where pervasive backtracking occurs. The prevalence of backtracking during infection can then be measured by determining how these RNAP occupancy differences in the presence and absence of Gre factors change in cells grown in broth versus infection.

**Fig 3:**
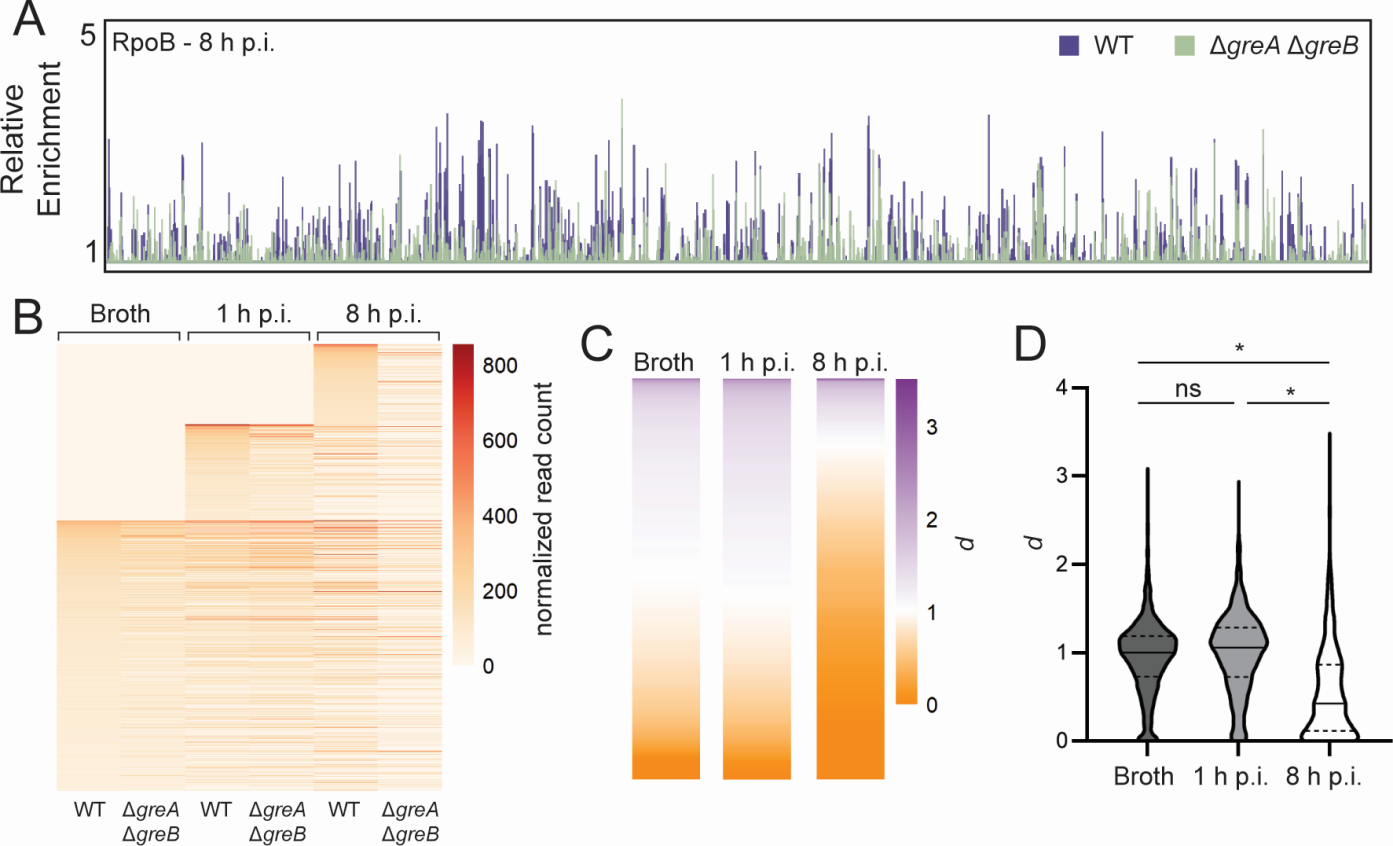
RNAP occupancy decreases in the absence of Gre factors. (**A**) Representative RNAP occupancy profile for wild-type (WT) cells or cells lacking Gre factors (Δ*greA* Δ*greB*) 8 h post-infection (p.i.) of HeLa cells as determined by PIC-seq of RpoB. Relative enrichment is defined as the ratio of IP and input read counts normalized to total read depth. (**B**) RNAP occupancy changes were visualized as a heat map, where every horizontal line represents the normalized read count for the same gene across each condition. All top transcribed genes for every condition combined are plotted (1,043 genes total, see Table S4). Each value represents the average of three independent replicates. Zeros are plotted for genes that do not arise in one condition but do arise in one or both of the other conditions. (**C**) Ratio *d* of normalized read count in the absence of Gre factors versus in the presence of Gre factors (WT) for top-transcribed genes in each condition averaged across three independent replicates. (**D**) Quantification of (**C**) showing ratio *d* calculated for every top-transcribed gene, where the median is shown by a solid line and quartiles by dashed lines. *P < 0.0001, ns: not significant, one-way ANOVA.

We measured RNAP occupancy in WT and Δ*greA* Δ*greB* cells grown in broth (LB-Lennox) and at 1 or 8 h post-infection (h p.i.) using PIC-seq (Table S3). Indeed, deletion of both Gre factors led to pronounced changes in RNAP occupancy genome wide (Fig. 3A; Fig. S4A and B). Given that inducing cell death during infection more than 100-fold by treatment with carbenicillin does not yield any significant difference in RNAP occupancy compared to WT cells (Fig. S2), the changes in RNAP occupancy we detect in the absence of Gre factors are unlikely to be due to any defects in cell survival. We found that RNAP occupancy generally decreases in the absence of the two Gre factors (Fig. 3A). This decrease is consistent with the activity of previously described accessory factors, such as helicases, that can remove stalled RNAPs from DNA (16, 23, 42–46). Backtracked RNAPs that are not rescued by the Gre factors are expected to be eventually removed by such helicases and other transcription terminators, leading to a decrease in RNAP occupancy.

Having determined that RNAP occupancy changes upon deletion of both Gre factors, we identified the genes that exhibited differential RNAP occupancy in each condition (broth, 1 h p.i., and 8 h p.i.). However, because cells grown in these conditions have widely different gene expression profiles, we could not always directly compare backtracking in the same gene across each condition. In other words, we could not compare backtracking at a gene that is transcriptionally active in one condition but not transcriptionally active in the other. Therefore, we categorized the genes by relative transcription level in each strain, within each condition. Relative transcription level was determined for each gene using the normalized number of reads mapping to that gene in the IP sample versus the input sample in the WT strain, averaged across three replicates. Genes were categorized independently for each condition (broth, 1 h p.i., and 8 h p.i.). This resulted in 631 top transcribed genes in broth, 645 at 1 h p.i., and 590 at 8 h p.i. (Fig. 3B; Table S4). A proportion of the top transcribed genes were found across all three conditions (283 genes) (Fig. S4C; Table S4). It is clear that RNAP occupancy differs at genes in the presence and absence of Gre factors, not only within each independent condition but also across conditions (Fig. 3B). These differences are still present at many of the 283 top transcribed genes that are shared between all three conditions (Fig. S4C and D).

**TABLE 1:** Gre factor-dependent RNAP occupancy changes in broth versus infection^*a*^

Transcription level	*d*_broth_	*d*_1 h p.i_.	*d*_8 h p.i_.	*d*′ 1 h p.i. versus broth	*d*′ 8 h p.i. versus broth
++	0.96	1.01	0.56	1.06	0.59
+++	0.91	0.99	0.52	1.09	0.57
++++	0.91	0.94	0.48	1.03	0.53
All genes	0.94	1.00	0.55	1.06	0.59

^*a*^ Ratio *d* was calculated as the normalized read counts mapping to a gene in the backtracking-prone state (Δ*greA* Δ*greB*) versus in WT for each condition and each transcription level (++, +++, and ++++). Ratio *d′* compares ratio *d* for 1 or 8 h p.i. to broth for each transcription level.

We performed a more quantitative analysis to attain an accurate picture of RNAP occupancy changes for cells grown during infection versus those grown in broth culture. We defined a ratio for differential RNAP occupancy, *d*, as the normalized read counts mapping to a gene in the backtracking-prone state (Δ*greA* Δ*greB*) versus those in WT. An equivalent ratio (*d* ≈ 1) indicates that there is little difference in RNAP occupancy independent of the presence or absence of the Gre factors. We interpreted these regions to be those that are not experiencing backtracking. A ratio that deviates from one (*d* < 1 or *d* > 1) indicates that there is a Gre factor-dependent difference in RNAP occupancy at the gene suggesting that these factors are playing an important role in maintaining RNAP progression at that gene. We interpreted these regions with *d* < 1 to be those that are experiencing significant RNAP backtracking. A ratio *d* > 1 indicates that Gre factors may normally be involved in suppressing RNAP occupancy at that gene. This ratio was calculated for every top-transcribed gene in each condition (Fig. 3C; Fig. S4E). For cells grown in broth and at 1 h p.i., the average ratio of RNAP occupancy in the absence of Gre factors versus in the presence is equivalent (0.94 and 1.00, respectively) (Table 1; Fig. 3D), suggesting that, on average, cells do not depend on Gre factors to maintain proper RNAP occupancy under these conditions and that backtracking is likely minimal. By contrast, cells residing within host cells, especially at 8 h p.i., do rely on the Gre factors to maintain RNAP occupancy (*d* = 0.55) (Table 1; Fig. 3D), suggesting that backtracking becomes pervasive as the infection progresses. Interestingly, this effect is independent of transcription level at 8 h p.i.; RNAP occupancy is abrogated in both lowly and highly transcribed genes.

We determined the extent to which backtracking is more prevalent during infection than broth. We accomplished this by quantifying the average differences in RNAP occupancy changes in broth versus infection. We defined a new ratio, *d′*, as the ratio of *d* for infection versus *d* for broth, *d′* =*d_inf_/d_broth_*. Compared to broth, backtracking is not more prevalent early in infection (at 1 h p.i.), but it is more prevalent at 8 h p.i., *d′* =0.59 (Table 1).

### Backtracking occurs within key genes necessary for *Salmonella* pathogenesis

**Fig 4:**
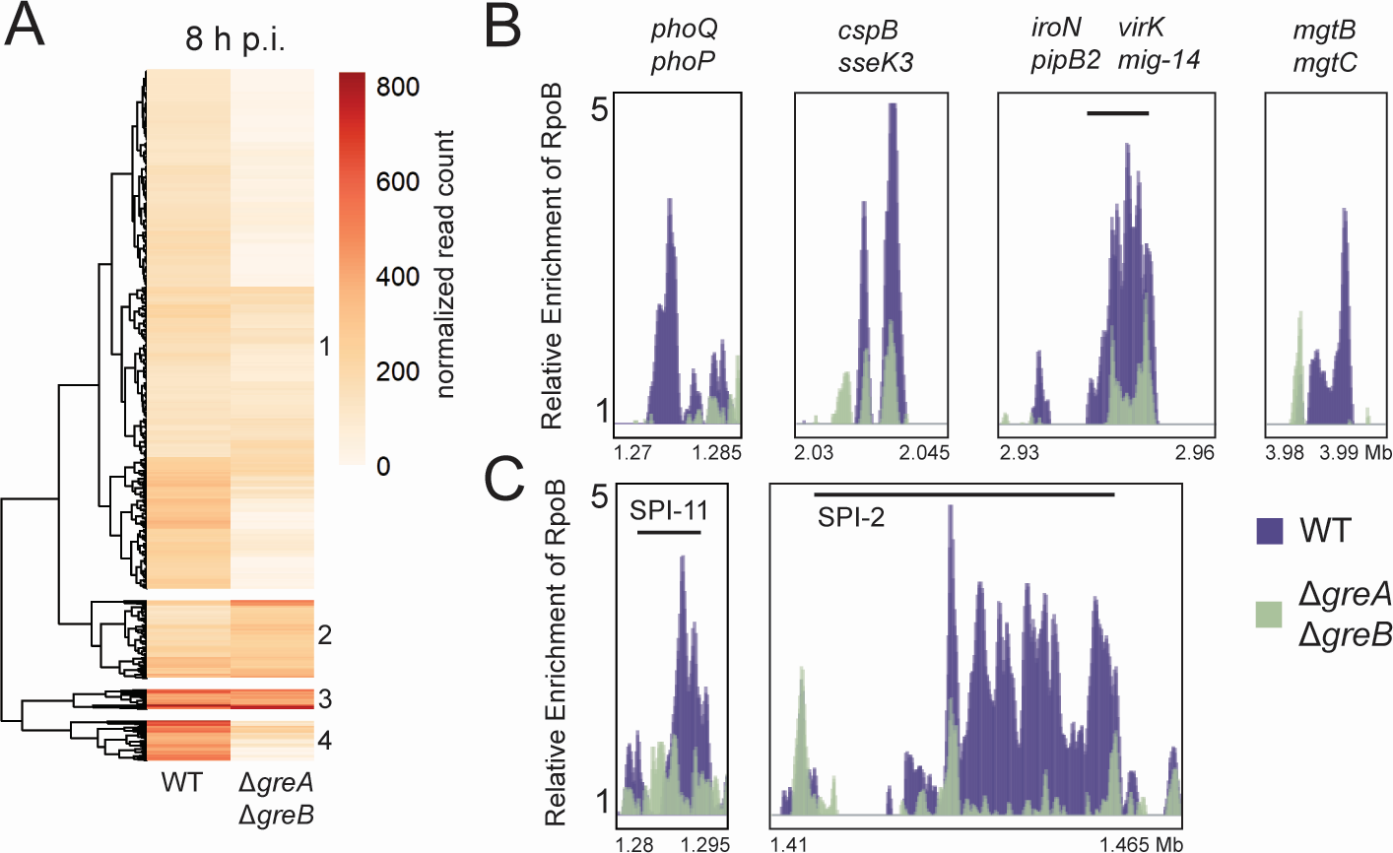
Backtracking is prevalent at key virulence genes during infection. (**A**) RNAP occupancy changes at 8 h post-infection (p.i.) in the absence of Gre factors visualized as a heat map. Each value represented is the average of three independent replicates. Hierarchical clustering (numbered 1–4) was performed using the pheatmap function in RStudio (see Table S5). (**B**) Key genes related to virulence in cluster four in (**A**) exhibit significant differences in RNAP occupancy (*P* < 0.05 for every gene listed, unpaired *t-*test). (**C**) Genes belonging to SPIs that fall into cluster one in (**A**) and exhibit significant differences in RNAP occupancy. Relative enrichment is defined as the ratio of IP and input read counts normalized to total read depth.

Having established that backtracking is more prevalent during infection genome wide, specifically at later timepoints, we endeavored to identify those regions where backtracking occurs and could potentially be problematic during infection. Thus, we focused on genes with the greatest changes in RNAP occupancy in the absence of Gre factors. These are the genes where RNAP activity is dependent on the function of the Gre factors, most likely due to backtracking. These were identified using hierarchical clustering (Fig. 4A; Fig. S5; Table S5 ).

As backtracking is minimal in broth and at 1 h p.i. (Fig. 3; Fig. S5), we focused on how genes were clustered by RNAP occupancy differences at 8 h p.i. (Fig. 4A). Genes at 8 h p.i. fell into four clusters based on similarities in RNAP occupancy changes in the presence and absence of Gre factors: cluster one, 466 genes; cluster two, 70 genes; cluster three, 18 genes; and cluster four, 36 genes (Fig. 4A; Table S5). We categorized the RNAP occupancy at genes within each of these hierarchical clusters as either Gre factor dependent or independent and identified enriched gene functions within each cluster.

In clusters four and one, RNAP occupancy is on average higher in the presence of Gre factors (WT) than in the absence (Δ*greA* Δ*greB*), indicating that the necessary levels of RNAP association with these genes depend on the presence of Gre factors. Genes in cluster four exhibit dramatic differences in RNAP occupancy depending on the presence of Gre factors, with an average *d* of 0.23. There was not enough statistical power to perform gene ontology (GO) functional enrichment analysis for genes in cluster four. However, manual annotation of these gene functions reveals that out of 36 genes, at least 17 have been previously implicated in *S*. Typhimurium virulence and/or pathogenesis (Fig. 4B; Table S5). RNAP occupancy at genes in cluster one also depends on the presence of Gre factors (*d* = 0.43), despite genes in cluster one having lower RNAP occupancy levels overall (Fig. 4A). According to GO functional enrichment analysis, cluster one genes are enriched for functions related to cellular and macromolecular localization, membrane biogenesis, regulation of metabolic processes, secretion, and translation. This cluster also consists of numerous SPI genes that exhibit large differences in RNAP occupancy when Gre factors are absent (Fig. 4C; Table S5). Of the 25 top-transcribed SPI-2 genes in this data set, 22 fall within cluster one. Similarly, all five of the top transcribed SPI-11 genes in this data set fall within cluster one. Efficient RNAP progression at these regions, and the virulence genes belonging to cluster four, would likely be critical to supporting the cell during infection, as these genes are required for cell survival throughout a significant proportion of the *S*. Typhimurium infection lifecycle (38). Our results suggest that infection of a host disrupts RNAP dynamics and that Gre factors are key to maintaining RNAP movement within these regions, and ultimately, supporting *S*. Typhimurium pathogenesis.

Genes in cluster two exhibit an average *d* ratio of 1.41, or greater RNAP occupancy in the absence than in the presence of Gre factors, indicating the RNAP occupancy at these genes does depend on the Gre factors, but in an opposite manner to those genes in clusters four and one. Genes in cluster two are enriched in functions related to cellular nitrogen compound biosynthetic processes (GO analysis). Cluster three includes genes where RNAP occupancy does not change in the absence of Gre factors (*d* = 0.99), indicating that these are Gre factor-independent genes; RNAP presence and/or activity at these genes does not depend on the Gre factors. Manual annotation of genes in cluster three reveals functions related to essential processes such as translation, carbon metabolism, and cell wall maintenance (Table S5).

Despite the functional enrichments we identified, the gene functions within each cluster vary. This is unsurprising, given our hypothesis that backtracking during infection is more prevalent due to stress from the host environment resulting in DNA damage within the pathogen. DNA damage and subsequent RNAP backtracking would occur randomly throughout the genome during infection, but would likely be concentrated in actively transcribed genes, based on recent models of DNA damage accumulation (24). Therefore, it tracks that the set of genes in which RNAP occupancy depends on Gre factors would be characterized by a wide range of gene functions.

In addition, we found that the prevalence of backtracking was not uniform genome wide. We reasoned that longer genes are more likely to accumulate DNA damage and therefore more likely to accumulate backtracked RNAPs. Accordingly, we found that the lengths of genes in cluster four, which exhibited the greatest differences in RNAP occupancy in a Gre factor-dependent manner, were significantly longer than the average length of all top-transcribed genes in this data set (Fig. S6). Overall, our results suggest that backtracking occurs at key virulence genes during infection and that Gre factors are critical for RNAP progression in these regions.

### Backtracking alters gene expression during infection

The observation that backtracking is more prevalent during infection than in broth culture suggests that proper expression of genes requires the resolution of backtracked RNAPs. In fact, prior work from others suggests that Gre factors are involved in the regulation of expression of a myriad of genes, including virulence genes, at least in broth culture (in minimal media (29) and in broth conditions that induce *S*. Typhimurium invasiveness (30)). However, whether this role is conserved and/or meaningful in the context of pathogenesis during infection of a host remained undefined. To examine whether the differential binding of RNAP we observe during infection due to the loss of both Gre factors ultimately correlates with altered gene expression, we compared the transcriptional profile of WT cells and cells lacking both Gre factors during infection using RNA-seq. We enriched *S*. Typhimurium from infected HeLa cells at 8 h p.i. and isolated total bacterial RNA. In parallel, we performed RNA-seq of WT and Δ*greA* Δ*greB* cells grown in broth (LB-Lennox). On average, approximately 14% of reads from each cDNA library from infection mapped to the *S*. Typhimurium genome, providing approximately 170× coverage (approximately 5.5 million aligned reads per sample).

**Fig 5:**
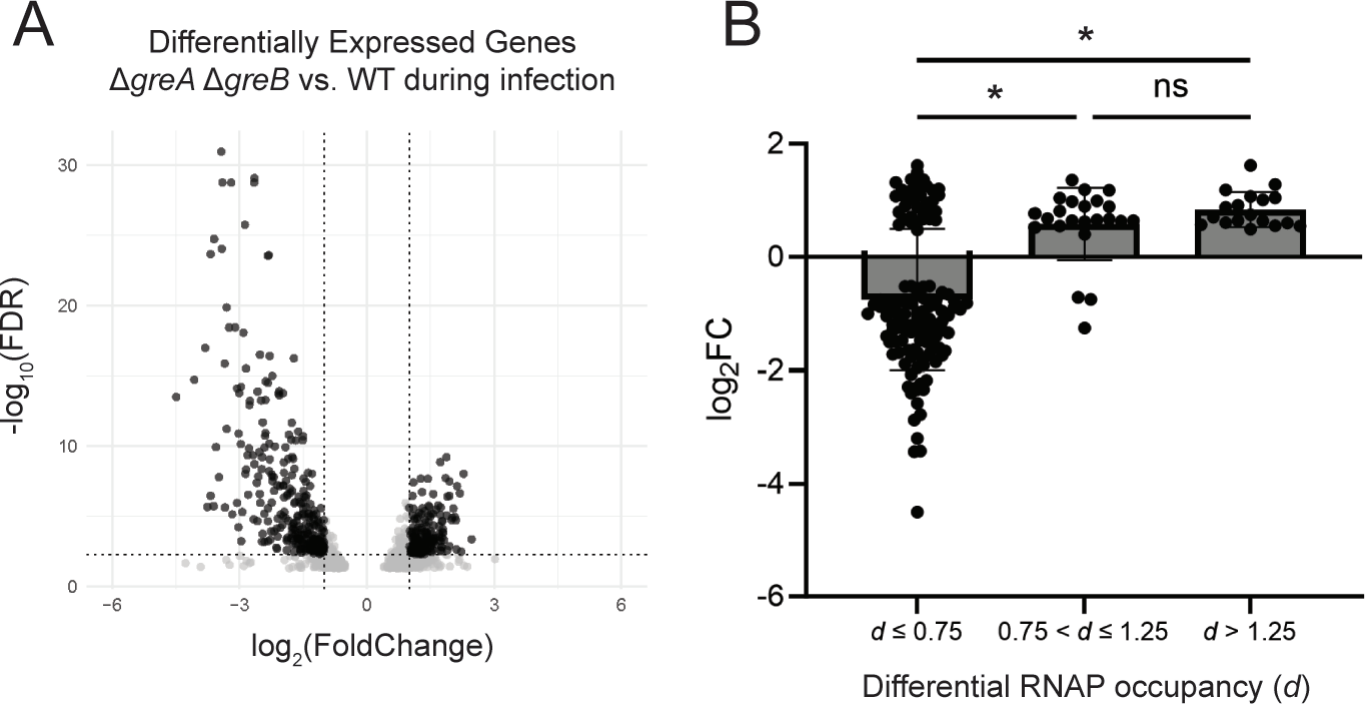
Effect of Gre factors on gene expression during infection. (**A**) Volcano plot of the distribution of differentially expressed genes in Δ*greA* Δ*greB* cells versus wild type (WT) during infection after DEseq2 analysis. FDR = false discovery rate (*P* value corrected for multiple testing). Each dot represents the mean value from three independent replicates of one gene. Black color indicates significant results (FDR < 0.05, log_2_FC > |1|) (**B**) Changes in gene expression for varying levels of backtracking severity. All top-transcribed genes at 8 h post-infection (see Table S4) were sorted into three groups by the value of their *d* ratio. The average change in gene expression (as indicated by log_2_ of the fold change) for each group was calculated. *P < 0.0001, ns: not significant, one-way ANOVA.

In total, 1,183 genes were differentially expressed in the absence versus the presence of Gre factors during infection (FDR-corrected *P* < 0.05) (Fig. 5A; Table S6). Expression levels for 713 of these genes differed by more than twofold (log_2_FoldChange ≥ |1|) (Table S6). Of these genes, 369 were downregulated and 344 were upregulated (Table S6). GO functional enrichment analysis of these differentially expressed genes reveals that expression of genes related to metabolic processes is significantly impacted by the Gre factors during infection, as these functions are overrepresented and underrepresented in downregulated and upregulated genes, respectively (Fig S7A). Interestingly, on average, SPI-1 genes were poorly expressed during infection in cells lacking Gre factors (Table S6), which is consistent with previous gene expression data from Δ*greA* Δ*greB* cells grown in broth culture (29, 30). This indicates that backtracking resolution facilitated by the Gre factors is likely required for efficient expression of these genes during infection, as was previously hypothesized (30). However, in contrast to previously reported results from cells grown in broth culture (29, 30), our data indicate that SPI-2 genes were moderately upregulated during infection in cells lacking Gre factors (Table S6). This contrast with previous studies is likely due to the difference in growth conditions, as multiple distinct pathways exist for SPI-2 induction that are triggered by different environmental conditions (47), such as those differences between broth culture and infection. Overall, our data suggest that the potential role of the Gre factors in regulating gene expression extends to infection as well.

During infection, we observe that RNAP occupancy decreases genome wide in the absence of both Gre factors. Changes in RNAP occupancy likely coincide with changes in gene expression. Indeed, we found a positive correlation between RNAP occupancy (as determined by PIC-seq of RpoB) and expression level (as determined by RNA-seq) for both WT and Δ*greA* Δ*greB* cells during infection (Pearson coefficient 0.53 and 0.55, respectively) (Fig. S7B), indicating that regions with low RNAP occupancy correlate with low gene expression. Accordingly, we hypothesized that genes experiencing RNAP backtracking during infection (a low *d* ratio) would also exhibit low gene expression. To test this, we again focused on genes at 8 h p.i., when backtracking is most prevalent genome wide. We categorized genes into three groups according to differential RNAP occupancy (*d* ≤ 0.75, 0.75 < *d* ≤ 1.25, and *d* > 1.25) and determined the average expression level (mean of the log_2_ of the fold change) of significantly differentially expressed genes (FDR < 0.05) within each group. Indeed, genes that experience severe backtracking (*d* ≤ 0.75) are significantly downregulated in the absence of the Gre factors compared to genes with little-to-no backtracking (0.75 < *d* ≤ 1.25), or those where the absence of Gre factors increases RNAP occupancy compared to WT (*d* > 1.25) (Fig. 5B). The data suggest that, on average, the expression of genes experiencing backtracking during infection in the absence of Gre factors is significantly decreased. Thus, not only does backtracking influence gene expression, but if backtracked RNAPs are not resolved, regulation of gene expression during infection suffers.

Interestingly, gene expression in cells lacking both Gre factors is affected more by a change in condition (infection versus broth culture) than gene expression in WT cells (Fig. S7C). For WT cells, a large proportion of genes do not exhibit significant changes in expression when comparing infection to broth. However, for cells lacking both Gre factors, there are very few genes whose expression does not change between conditions, suggesting that cells lacking both Gre factors are more sensitive to the change in condition than WT cells.

### Resolution of backtracking is critical to pathogenesis

**Fig 6:**
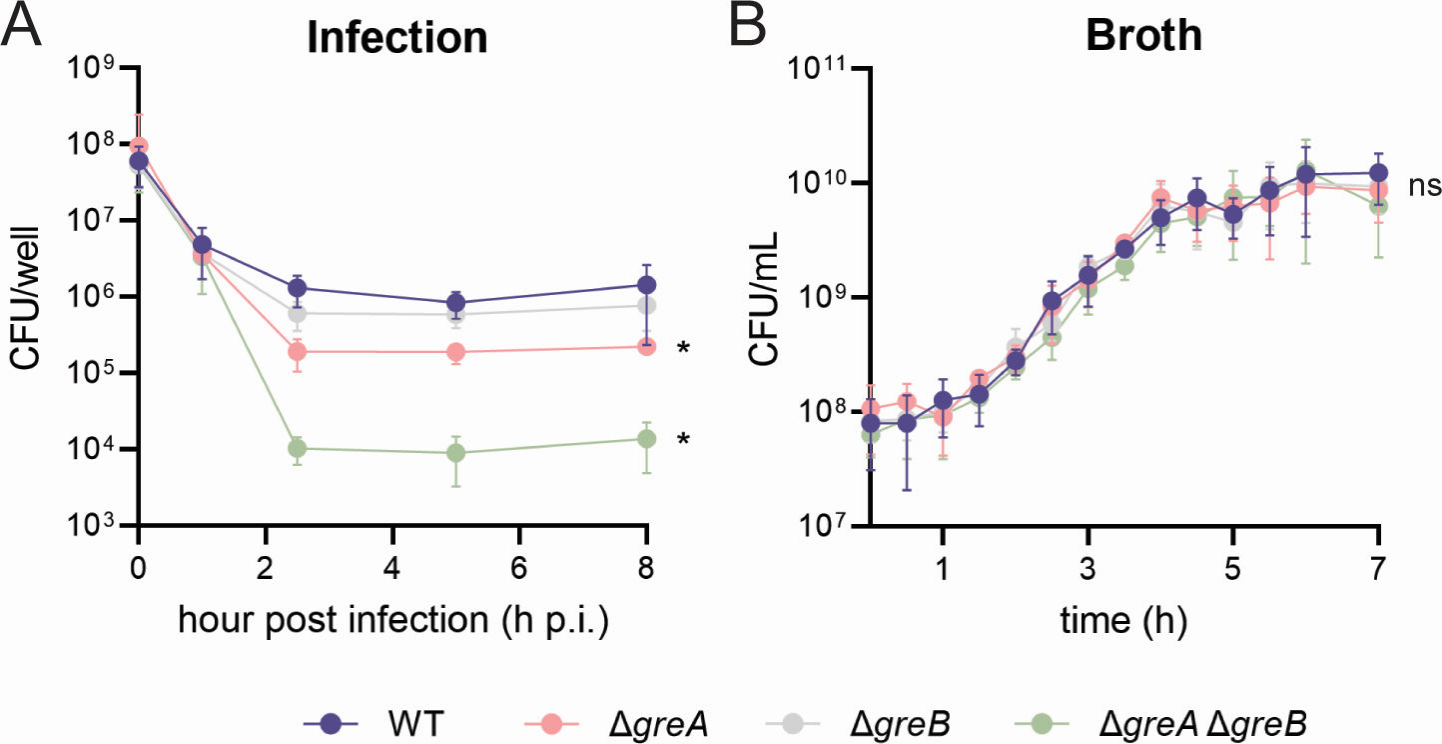
Resolution of backtracking during infection is key to pathogenesis. Average number of colony forming units (CFUs) of backtracking-prone cells (**A**) isolated during infection or (**B**) cultured in LB-Lennox culture at 37°C. Error bars indicate the standard deviation of at least six independent replicates. *P < 0.005, ns: not significant, one-way ANOVA with Bonferroni correction.

Knowing that backtracking is prevalent at key virulence genes during infection and that genes experiencing backtracking are downregulated, we tested whether disruption to RNAP progression is problematic for pathogenesis in general. A prior study demonstrated that the ability of *S*. Typhimurium lacking both Gre factors to invade human host cells or colonize a mouse spleen was ablated compared to WT (30). However, how backtracking affected the growth of *S*. Typhimurium over the course of the infection, beyond the initial host invasion, was not examined. To determine the impact of pervasive backtracking on *S*. Typhimurium pathogenicity throughout the course of infection, we infected HeLa cells with backtracking-prone *S*. Typhimurium lacking one or both Gre factors. We measured the number of viable intracellular *S*. Typhimurium over the course of the infection using a standard gentamicin protection assay (Fig. 6). Deletion of *greB* alone did not significantly affect the growth of *S*. Typhimurium inside HeLa cells over the course of the infection, while deletion of *greA* led to a modest, yet statistically significant loss in intracellular growth. However, deletion of both Gre factors significantly abrogated intracellular growth of *S*. Typhimurium throughout the duration of the infection (Fig. 6A). Interestingly, at 1 h p.i., there are no differences in the viability of cells in the presence or absence of Gre factors, supporting our earlier finding that backtracking is not pervasive at this point during the infection. The negative effect of depleting cells of both Gre factors is specific to infection, as there were no significant differences in the growth of these strains compared to WT when cultured in LB-Lennox (Fig. 6B). Taken together, our data indicate that pervasive backtracking during infection disrupts gene expression, and resolution of that backtracking is critical to intracellular survival.

## DISCUSSION

During infection, bacterial pathogens must survive numerous host defense mechanisms that are not only damaging to the cell itself but can also lead to DNA damage capable of stalling RNAP (14–17). Pathogens must quickly sense and respond to the host environment, and any disruption to these virulence gene programs at the transcriptional level would be significant. Our work suggests that infection of a host significantly impacts bacterial RNAP dynamics and gene expression, causing pervasive backtracking of these machinery along the bacterial chromosome that must be resolved to ensure successful pathogenesis.

Our data show that disruptions to RNAP movement increase as the infection progresses. As evidenced by the lack of difference in RNAP occupancy in the presence and absence of Gre factors at 1 h p.i. (Fig. 3; Table 1), it appears that early during infection, RNAPs are not yet perturbed by interactions with the host. This is further emphasized by the growth of cells at 1 h p.i.: cells at this timepoint do not require Gre factors to survive during infection (Fig. 5A). It is possible, however, that RNAP stalling at 1 h p.i. is underestimated in our experiments due to detection thresholds of PIC-seq as a population assay and/or the heterogeneity of the cell population at this early point post-infection (48). Cells at 8 h p.i., however, have endured stress from the host environment for much longer than those at 1 h p.i., potentially increasing the likelihood of DNA damage accumulation that can stall RNAPs and lead to backtracking. Accordingly, cells at this later timepoint exhibit large differences in RNAP occupancy and disruptions to gene expression. These observations, combined with the lack of intracellular viability observed in the absence of Gre factors, suggest that cells require backtracking resolution for intracellular proliferation, most significantly later during infection. These problems are likely occurring at earlier timepoints; however, they are most apparent at later timepoints.

In addition to perturbing transcription, an increase in the prevalence of backtracking during infection could have significant impacts on genome integrity. Transcription is an obstacle to DNA replication due to the lack of spatiotemporal separation between the two processes (49). These conflicts pose significant threats to genome stability regardless of directionality (49), and their occurrence could be worsened during infection due to the increased prevalence of backtracked RNAPs (41). Specifically, co-directional conflicts between a backtracked RNAP and an oncoming replication fork lead to double-stranded breaks in the DNA (41). *S*. Typhimurium cells lacking key conflict resolution factors, such as UvrD or Rep helicases (50), exhibit significant growth defects during infection of HeLa cells (Fig. S8), suggesting that conflicts could be an issue for pathogens during infection. However, further work is required to determine the extent to which replication-transcription conflicts occur in pathogens during infection and whether increased backtracking during infection exacerbates these conflicts. Previous work has also shown that cells that are prone to backtracking (lacking either Gre factor) accumulate mutations faster (41) and have higher rates of recombination than cells with both Gre factors (51). In addition, stalled RNAPs trigger downstream DNA repair pathways that can be mutagenic (14, 22–26). Although pathogenic cells normally carry both Gre factors and will thus experience less backtracking under regular conditions, the fact that these RNAP stalling events are much more prevalent during infection inherently increases the chances of mutagenic events occurring. Therefore, we postulate that, though the host is fighting the bacteria with DNA-damaging mechanisms such as oxidative stress, the host may also be enabling bacteria to attain mutations that could lead to adaptive evolution. This model requires further investigation that is outside of the scope of the work presented here. However, it is certainly interesting to investigate in future studies as this dichotomy could perpetuate the development of AMR and/or hypervirulence.

Our findings show for the first time that backtracking occurs genome wide in a bacterial pathogen during infection to a greater extent than in the same cells grown outside the host environment (LB-Lennox broth culture, in our case). Thus, backtracking resolution facilitated by the Gre factors is likely to be even more important during infection due to the increased prevalence of these RNAP disruptions. Consistent with this, even though Gre factor-dependent expression of a variety of genes had been identified in broth culture models (i.e., outside of infection) (29, 30), our data demonstrate that the role of the Gre factors in regulating gene expression does indeed extend to cells grown within the host environment during infection. Specifically, our data show the downregulation of genes that experience backtracking. Interestingly, in *S*. Typhimurium grown in minimal broth culture, Gre factors had been previously shown to facilitate transcriptional elongation of central metabolic genes that resist oxidative stress (29). Our data extend these findings and reveal that backtracking occurs within three of these metabolic genes (*nuoA*, *eno*, and *gapA*) at 8 h p.i. (Table S4), suggesting that, during infection, the ability of the Gre factors to facilitate transcriptional elongation of key metabolic genes may extend to infection as well. In light of these previously published results, our data are not mutually exclusive with the possibility that some of the changes in RNAP occupancy that we observe during infection are due to altered metabolism within the pathogen itself, and not solely due to damaging host defense mechanisms. In other words, in much the same way that different broth conditions alter *S*. Typhimurium metabolism (29), infection of a host may lead to changes in endogenous *S*. Typhimurium metabolism (such as generation of ROS) that could affect RNAP backtracking. Regardless of the origin of any increased damage to the DNA in *S*. Typhimurium during infection, our data nonetheless suggest that RNAP backtracking is worsened during infection as a result.

The pervasive, genome-wide backtracking during infection that we identified likely impacts a multitude of gene functions. Therefore, our work supports the possibility that Gre factors are required for backtracking resolution within a wide variety of genes and determinants of pathogenesis, some of which may have yet to be defined. Overall, our results highlight an emerging appreciation for the role of the Gre factors in bacterial pathogens, specifically during infection. This class of proteins promotes survival and guarantees proper gene expression during infection by regulating and modulating RNAP backtracking during transcription elongation. In fact, similar impacts of the Gre factors on virulence phenotypes have also been shown in other, highly divergent bacterial species, highlighting the conservation of the importance of Gre factors for pathogenesis (52–54).

Precise regulation of gene expression during infection is critical to the survival of bacterial pathogens. Our data strongly suggest that host defense mechanisms, such as those resulting in DNA damage, threaten this precision. Any disruptions to RNAP progression arising during infection must be resolved. Altogether, our results support the model that backtracking is prevalent during infection and that Gre factors are required to ensure proper gene regulation and survival during infection.

## MATERIALS AND METHODS

### Bacterial strains and growth conditions

*Salmonella enterica* serovar Typhimurium SL1344 was the WT strain used in these studies. Derivative mutant strains are listed in Table S7. Bacteria were grown on LB-Lennox agar plates at 37°C with the following antibiotics when appropriate: 25 µg/mL chloramphenicol or 50 µg/mL kanamycin. Single colonies were used to inoculate liquid cultures in LB-Lennox (5 g/L yeast extract, 10 g/L tryptone, 5 g/L NaCl) and grown at 37°C with aeration (260 rpm).

### Construction of chromosomal deletion mutants

The bacteriophage λ Red recombination system was used for the construction of chromosomal deletion mutants (55, 56). The plasmid pSIM27 was transformed into WT SL1344 for expression of the Red recombinase system. Kanamycin and chloramphenicol resistance cassettes were amplified with Phusion High-Fidelity polymerase (Thermo) from T-SACK gDNA (57) using primers that contained 40 nucleotides homologous to the regions flanking the *greA* or *greB* genes (including the ATG start codon, Table S7). Amplicons were electroporated separately into SL1344 and cells were grown on selective LB agar to yield single knockout strains Δ*greA* (*greA::*Kan, HM4527) and ΔgreB (*greB::*Cat, HM4525). To make the double knockout strain Δ*greA* Δ*greB* (HM4529), the *greA* gene was replaced with the kanamycin resistance cassette in the ΔgreB strain as above. Every deletion was confirmed by Sanger sequencing.

### Mammalian cell culture

HeLa cells (ATCC) were grown in high glucose Dulbecco’s minimal essential medium (DMEM, Gibco – 11995065) supplemented with 10% heat-inactivated fetal bovine serum (FBS, R&D Systems), 4 mM L-glutamine (Gibco), and 1X Penicillin/Streptomycin (Gibco). Antibiotic-free media of the same formulation was used for bacterial infections. Cells were maintained at 37°C with 5% CO_2_ and passaged following ATCC guidelines.

### Seeding and bacterial invasion of mammalian cells

HeLa cells at low passage numbers were seeded at the following densities 16–18 h before infection: 1.5 × 10^5^ cells per well (24-well dish) or 1.14 × 10^7^ (15 cm plate). Immediately prior to infection, HeLa cells were washed with 1× phosphate-buffered saline (PBS; Gibco), and antibiotic-free media was added to the cells. Cultures of *S*. Typhimurium were inoculated in LB from single colonies and grown overnight at 37°C while shaking. The next day, the precultures were diluted back to OD_600_ = 0.05 in LB and grown at 37°C while shaking until OD_600_ reached 0.6 (approximately 2 h). Bacteria were collected by centrifugation, washed in 1× PBS, resuspended in antibiotic-free media, and used immediately to infect HeLa cells at an MOI of ~100:1. Bacteria were allowed to invade for 1 h at 37°C with 5% CO_2_. Bacteria were removed after invasion, and HeLa cells were washed once with 1× PBS and fresh antibiotic-free media was added. Thirty minutes later [1.5 h post-infection (p.i.)], gentamicin (Gibco) was added to a final concentration of 50 µg/mL for the duration of the experiment. Infected HeLa cells were maintained at 37°C with 5% CO_2_ until the times indicated below.

### Gentamicin protection assays

HeLa cells were seeded in 24-well dishes and infected with bacteria as described above. Extracellular growth of *S*. Typhimurium was inhibited by gentamicin as described above. At indicated timepoints, infected HeLa cells were washed once with 1× PBS and lysed with ice-cold 1% Triton X-100 in H_2_O. Viable bacteria were enumerated by plating on LB agar and grown at 37°C overnight. A one-way ANOVA was performed at 2.5, 5, and 8 h p.i. timepoints with Bonferroni correction to determine statistical significance.

### Chromatin immunoprecipitation from infection

HeLa cells were seeded in 15 cm plates and infected with bacteria as described above. For treatment with rifampicin, 1 mg/mL (final concentration) was added to the media 10 min prior to each timepoint. At indicated timepoints, media was removed from infected HeLa cells, the cells were washed once with 1× PBS and then crosslinked in 1% methanol-free formaldehyde (Thermo) in 1× PBS for 10 min at room temperature. Crosslinking was subsequently quenched with 0.5 M glycine. Crosslinked infected HeLa cells were washed twice with 1× PBS, then dislodged in 1× PBS by scraping. Cells from two 15 cm plates were combined for each replicate and collected by centrifugation. Cell pellets were stored at −80°C for future processing. Pellets were thawed on ice and resuspended in 2.5 mL ice-cold NPT lysis buffer (58) [50 mM Tris–HCl pH 7.5, 150 mM NaCl, 5 mM ethylenediaminetetraacetic acid (EDTA), 0.5% NP-40, 0.1% Triton X-100, complete protease inhibitor cocktail (Roche) added fresh] for 10 min on ice. Lysozyme was added to 10 mg/mL and the lysate was incubated at 37°C for 30 min. Lysates were sonicated for 5 cycles of 30 s on/off (2.5 min total sonication time) at 4°C in a Bioruptor Plus sonication system (Diagenode) and pelleted by centrifugation at 8,000 rpm for 15 min at 4°C. A 40 µL aliquot was taken from the lysate supernatant as the input control. For the immunoprecipitation, 6 µL RpoB monoclonal antibody (8RB13, Thermo) was added to the lysate supernatant and rotated overnight at 4°C. The next day, 90 µL of a 50% protein A Sepharose bead slurry (GE) was added, and IPs were incubated for 1 h at room temperature with gentle rotation. Beads were pelleted by centrifugation at 2,000 rpm for 1 min. The supernatant was discarded, and the beads were washed six times for 3 min each in wash buffer (50 mM Tris-HCl pH 7.0, 150 mM NaCl, 5 mM EDTA, 1% Triton X-100), followed by one wash with TE pH 8.0. The elution was carried out at 65°C for 10 min in 200 µl elution buffer I (50 mM Tris pH 8.0, 10 mM EDTA, 1% SDS). Beads were pelleted at 5,000 rpm for 1 min and the supernatant was saved. The beads were washed with 150 µl elution buffer II (10 mM Tris-HCl pH 8.0, 1 mM EDTA, 0.67% SDS) and pelleted at 7,000 rpm for 1 min. The second supernatant was combined with the first eluate. The combined eluates and the accompanying input controls were de-crosslinked overnight by incubation at 65°C. The following day, the eluates and input controls were treated with proteinase K (0.4 mg/mL) at 37°C for 2 h. Sodium acetate was added and the DNA was purified by phenol:chloroform:isoamyl alcohol extraction. The DNA was precipitated in 100% ethanol at −20°C and pelleted before being resuspended in resuspension buffer (10 mM Tris-HCl pH 8.0, 1 mM EDTA).

### Chromatin immunoprecipitation from broth

Cultures of *S*. Typhimurium were inoculated in LB from single colonies and grown overnight at 37°C while shaking. The next day, the precultures were diluted back to OD_600_ = 0.05 in LB and grown at 37°C while shaking until OD_600_ reached 0.6 (approximately 2 h). Bacteria were crosslinked with 1% formaldehyde for 20 min at room temperature before being quenched with 0.5 M glycine. Cells were collected by centrifugation and washed once in cold 1× PBS. Cell pellets were resuspended in 1.5 mL Solution A (10 mM Tris–HCl pH 8.0, 20% wt/vol sucrose, 50 mM NaCl, 10 mM EDTA, 10 mg/mL lysozyme, and 1 mM AEBSF) and incubated at 37 ° C for 30 min. After incubation, 1.5 mL of 2× IP buffer (100 mM Tris pH 7.0, 10 mM EDTA, 2% Triton X-100, 300 mM NaCl, and 1 mM AEBSF, added fresh) was added and lysates were chilled on ice for 30 min. Lysates were sonicated four times for 10 s (40 s total sonication time) at 30% amplitude and pelleted by centrifugation at 8,000 rpm for 15 min at 4°C. A 40 µL aliquot was taken from the lysate supernatant as the input control. For the immunoprecipitation, 2 µL of RpoB monoclonal antibody (clone 8RB13, Thermo) was added to 1 mL of the lysate supernatant and rotated overnight at 4°C. The next day, 30 µL of 50% protein A Sepharose bead slurry (GE) was added, and IPs were incubated for 1 h at room temperature with gentle rotation. Beads were pelleted by centrifugation at 2,000 rpm for 1 min. The supernatant was discarded, and the beads were washed six times for 3 min each in wash buffer (50 mM Tris-HCl pH 7.0, 150 mM NaCl, 5 mM EDTA, 1% Triton X-100), followed by one wash with TE pH 8.0. The elution was carried out at 65°C for 10 min in 100 µL elution buffer I (50 mM Tris pH 8.0, 10 mM EDTA, 1% SDS). Beads were pelleted at 5,000 rpm for 1 min and the supernatant was saved. The beads were washed with 150 µL elution buffer II (10 mM Tris-HCl pH 8.0, 1 mM EDTA, 0.67% SDS) and pelleted at 7,000 rpm for 1 min. The second supernatant was combined with the first elution. The combined eluates and the accompanying input controls were de-crosslinked overnight by incubation at 65°C. The following day, the eluates and input controls were treated with proteinase K (0.4 mg/mL) at 37°C for 2 h. Sodium acetate was added and the DNA was purified by phenol:chloroform:isoamyl alcohol extraction. The DNA was precipitated in 100% ethanol at −20°C for 1 h and pelleted, before being resuspended in resuspension buffer (10 mM Tris-HCl pH 8.0, 1 mM EDTA).

### PIC deep sequencing and data processing

Sequencing libraries were prepared using a Nextera XT DNA Library Preparation kit (Illumina). Libraries were deep sequenced by the Vanderbilt Technology for Advanced Genomics (VANTAGE) sequencing core (Vanderbilt University) on an Illumina NovaSeq platform, resulting in approximately 24M × 150 bp paired-end reads per sample. Raw reads were trimmed (Trimmomatic v0.39 (59)) and then mapped to the *S. enterica* serovar Typhimurium strain SL1344 genome (GenBank: FQ312003.1) using Bowtie2 v2.2.5 (60). Both PCR and optical duplicates were removed using Picard v1.3 (Broad Institute) and bam files were sorted and indexed using SAMtools v1.13 (61).

The number of reads mapping to each gene in both the IP and input samples was quantified using featureCounts v2.0.3 (62) and then normalized to the total mapped reads. For each gene, the normalized number of reads in the input sample was subtracted from the normalized number of reads in the IP sample and averaged across three independent replicates to calculate the “normalized read count.” For any gene where this calculation resulted in a negative number, the normalized read count was redefined as 0 for that gene. At this point, rDNA genes were excluded from further analysis. Genes were then categorized into five groups based on transcription level using K-means clustering for each individual condition. Transcription level was defined by the normalized read count in the wild-type sample for each condition. The three groups containing the most transcribed genes for each condition were used for further analysis. Ratio *d* was calculated for these genes by dividing the normalized read count for the Δ*greA* Δ*greB* strain by the normalized read count in the wild-type strain. Ratio *d′* was calculated by dividing *d* from the 1 h p.i. or 8 h p.i. condition by the *d* from the broth condition. Heatmaps were plotted and hierarchical clustering was performed using the pheatmap package in RStudio. Gene ontology functional enrichment analysis was performed using the PANTHER v.14 pipeline (63) through The Gene Ontology Resource (64, 65).

For enrichment analysis, peaks were called from processed bam files using macs2 v.2.2.7.1 (66) and assigned to genome features using the “closest” tool from the BEDTools suite v.2.30.0 (67).

To visualize relative enrichment, bam files were normalized to the total number of mapped reads, and the ratio of IP versus input read depths was calculated using the bamCompare tool from the deepTools suite v3.5.1 (68) to generate bedgraph files. These bedgraph files were visualized on the Integrated Genomics Viewer (igv) platform v2.12.1 (69).

All statistical tests were performed in GraphPad Prism v9.

### Bacterial RNA isolation

HeLa cells were seeded and infected with *S*. Typhimurium (12 15 cm plates for WT and 24 15 cm plates for Δ*greA* Δ*greB*) as described above. Bacterial RNA was isolated from infected HeLa cells essentially as described previously (36). Briefly, HeLa cells were washed once with pre-chilled 1X PBS after 8 h p.i. and lysed on ice in 15 mL lysis buffer (0.1% SDS, 1% acidic phenol, 19% ethanol in water, ice cold), which also served to stabilize the RNA. Lysates from each plate were collected by scraping and pooled together. Bacteria were collected from the lysates by centrifugation at 3,300× *g* for 30 min at 4°C. The pellet was washed three times in wash buffer (0.1% acidic phenol, 19% ethanol in water, ice cold) and centrifuged at 3,800× *g* for 30 min at 4°C each time. After the final wash, the bacteria were resuspended in <1 mL wash buffer, moved to a 1.5 mL microcentrifuge tube (bacteria across HeLa lysates for each *S*. Typhimurium genotype were combined at this point), and collected by centrifugation at 16,000× *g* for 2 min at 4°C. The pellet was resuspended in 1 mL TRIzol on ice by pipetting up and down ~60 times. The resuspended pellet was stored at −80°C. Total RNA was extracted as previously described (70) and stored at −80°C prior to DNase treatment (below). The infection process and RNA isolation for library preparation/deep sequencing were performed in duplicate, yielding two independent replicates for both WT and Δ*greA* Δ*greB* strains.

In parallel, RNA was extracted from WT and Δ*greA* Δ*greB S*. Typhimurium grown in broth culture (LB-Lennox) as a control. Overnight cultures grown from single colonies were diluted back to OD_600_ = 0.05 in LB-Lennox and grown at 37°C while shaking. Once the culture reached an OD_600_ of 0.6., 1.5 mL of cells were mixed with 1.5 mL of 100% ice-cold methanol and incubated on ice for 10 min. Bacteria were collected by centrifugation at 4,000 rpm for 5 min and immediately used for total RNA extraction using the GeneJET RNA Purification Kit (Thermo) according to the manufacturer’s instructions. Purified RNA was stored at −80°C prior to DNase treatment (below). RNA was isolated for library preparation/deep sequencing from three independent replicates for both WT and Δ*greA* Δ*greB* strains.

A 1 µg sample of RNA from each condition (two replicates/infection, three replicates/broth) was treated with RNase-free DNase I for 40 min at 37°C. The digestion was quenched by the addition of EDTA and incubation at 65°C for 10 min. RNA was stored at −80°C prior to library preparation.

### cDNA library preparation, deep sequencing, and data processing

Ribo-depletion, library preparation, and deep sequencing were all performed by the VANTAGE Sequencing Core (Vanderbilt University). DNase-treated total RNA samples (500 ng) were depleted of rRNA using NEBNext rRNA Depletion Kit [New England Biolabs (NEB)]. Libraries were prepared using the NEBNext Ultra II Directional RNA Library Prep Kit for Illumina (NEB) with indices from NEBNext Multiplex Oligos for Illumina (NEB). Libraries were deep sequenced on an Illumina NovaSeq platform, resulting in approximately 50M × 150 bp paired-end reads per sample.

Raw reads were trimmed [Trimmomatic v0.39(59)] and mapped to the *S. enterica* serovar Typhimurium strain SL1344 genome (GenBank: FQ312003.1) using Bowtie2 v2.2.5 (60). Both PCR and optical duplicates were removed using Picard v1.3 (Broad Institute) and bam files were sorted and indexed using SAMtools v1.13 (61). The number of reads mapping to each gene was quantified using featureCounts v2.0.3 (62), from which transcripts per million (TPM) were calculated by hand. Differential expression analysis of loci with more than 10 mapped reads total across all samples was performed with DEseq2 v1.38.3 (71) in RStudio v4.2.3. Plots were created using ggplot2 v3.4.3 in RStudio or GraphPad Prism v9. Gene ontology functional enrichment analysis was performed using the PANTHER v.14 pipeline (63) through The Gene Ontology Resource (64, 65).

## Data Availability

Datasets generated during this study are deposited within NCBI BioProject, accession number: PRJNA1038755.

## SUPPLEMENTAL MATERIAL

Supplemental Material mbio.02737-23-s0001.docx Figures S1-S8, Tables S1 and S2, and captions for Tables S3-S6.

Table S3 mbio.02737-23-s0002.xlsx PIC-seq summary.

Table S4 mbio.02737-23-s0003.xlsx Top transcribed genes in cells grown in broth.

Table S5 mbio.02737-23-s0004.xlsx RNAP occupancy changes for the top transcribed genes as categorized by hierarchical clustering.

Table S6 mbio.02737-23-s0005.xlsx RNA-seq summary.

ASM does not own the copyrights to Supplemental Material that may be linked to, or accessed through, an article. The authors have granted ASM a non-exclusive, world-wide license to publish the Supplemental Material files. Please contact the corresponding author directly for reuse.
