# Supplementary material for: Pathogenic bacteria experience pervasive RNA polymerase backtracking during infection
Source: mBio. 2023 Dec 14;15(1):e02737-23. doi: 10.1128/mbio.02737-23 (PMC10790778; doi:10.1128/mbio.02737-23)
Supplement: Supplemental Material — Figures S1-S8, Tables S1 and S2, and captions for Tables S3-S6. [file mbio.02737-23-s0001.docx]

**Supplementary Material**

**
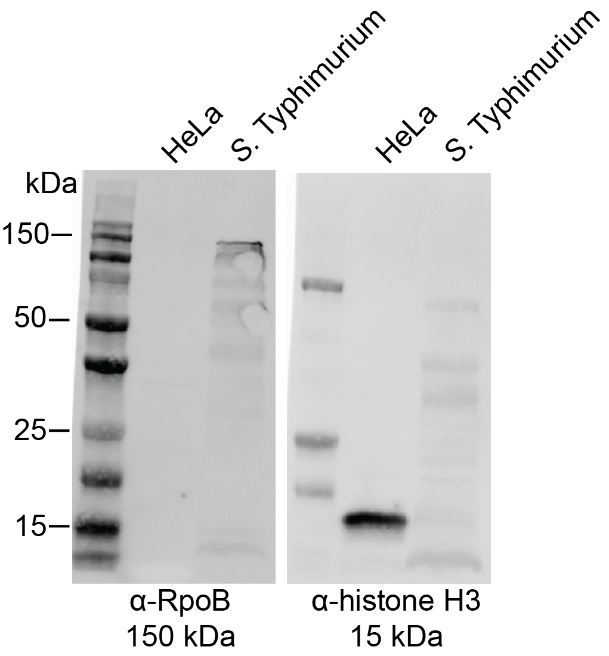
**

**S1 Figure. RpoB antibody is specific to bacterial protein.** Western blot showing that the native RpoB antibody used in these studies does not cross-react to proteins specific to HeLa cells. HeLa whole cell lysate was prepared in RIPA buffer and diluted into Laemmli sample buffer. *S.* Typhimurium whole cell lysate was prepared in lysis buffer (10 mM Tris-HCl pH = 7, 10 mM EDTA, 1X protease inhibitor, 0.1 mg/mL lysozyme) and diluted into Laemmli sample buffer. Lysates were boiled for 10 min and loaded onto a 12% Mini-PROTEAN TGX gel (Bio-Rad). Proteins were subsequently transferred to a PVDF nitrocellulose membrane (Bio-Rad). The membrane was blocked for 1 h at room temperature with Intercept PBS blocking buffer (LI-COR) before being immunoblotted with anti-RpoB (8RB13, Thermo) and anti-Histone H3 (PA5-16183, Thermo) antibodies overnight at 4°C. The blot was incubated with secondary antibodies IRDye-680RD and 800CW (LI-COR) for 30 min at room temperature before being imaged on an Odyssey Imager (LI-COR).


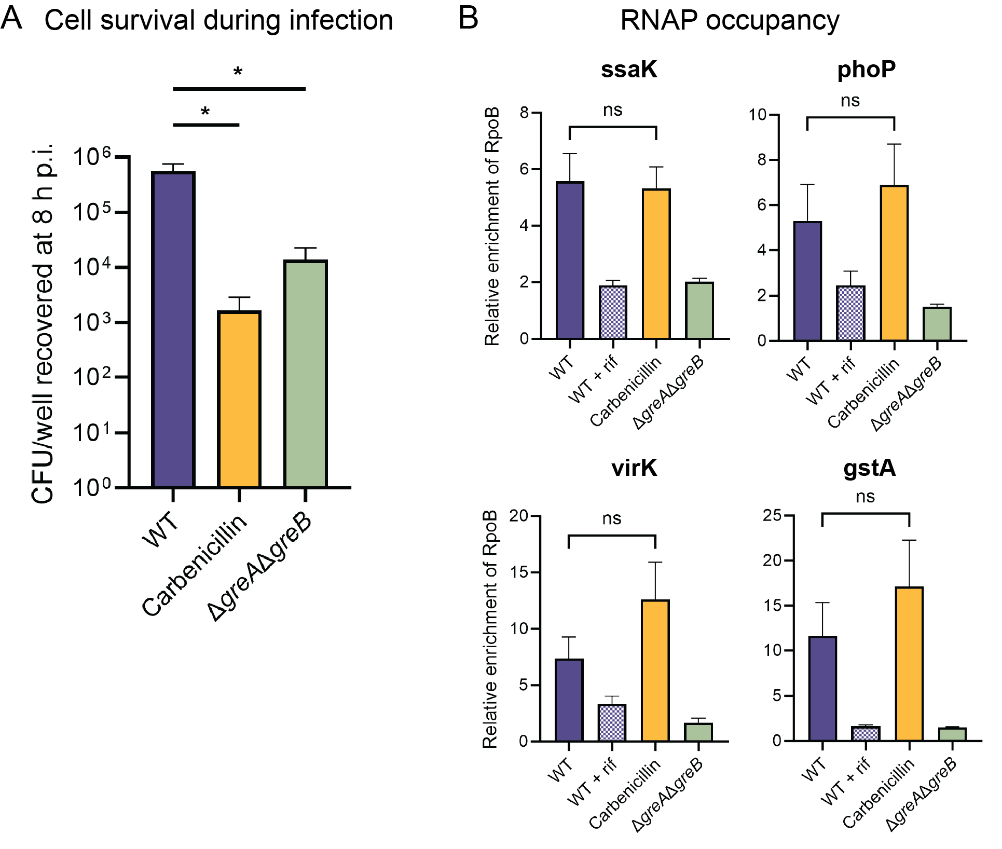


**S2 Figure. Changes to RNAP occupancy in the absence of Gre factors occur independently of loss in cell viability.** (A) Colony forming units (CFUs) of WT *S.* Typhimurium, WT cells in the presence of carbenicillin, and *S.* Typhimurium cells lacking Gre factors at 8 h post infection (p.i.) of HeLa cells as determined by gentamicin protection assay. Carbenicillin was added to a final concentration of 6.4 mg/mL at 1 h p.i. Data represent the means and standard deviation of at least six independent replicates. **p*<0.0001, one-way ANOVA. (B) RNAP occupancy was determined by PIC-qPCR at the indicated genes at 8 h p.i.. Where indicated, the antibiotic rifampicin (rif) was added ten minutes prior to crosslinking (see methods). Relative enrichment is defined as the ratio of gene copy number of the indicated gene to gene copy number of *eutN.* Data represent the means and SEM of at least four independent replicates. ns: not significant, one-way ANOVA.


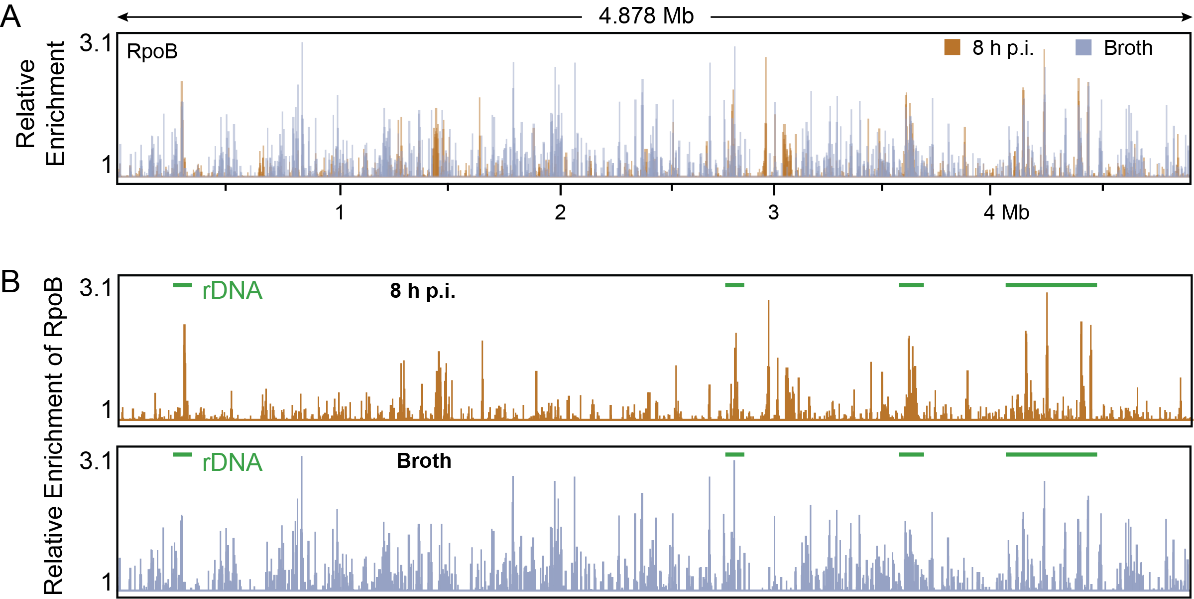


**S3 Figure. RpoB ChIP-seq signal from broth and infection.** (A) Representative overlay of *S.* Typhimurium RNAP occupancy as determined by PIC-seq of RpoB (the beta subunit of RNAP) 8 h post infection (p.i.) of HeLa cells or from cells grown in broth culture. (B) Reads mapped to the *Salmonella* genome display areas of high RNAP enrichment at regions of rDNA in both conditions (green bars). Relative enrichment is defined as the ratio of IP and total read counts.


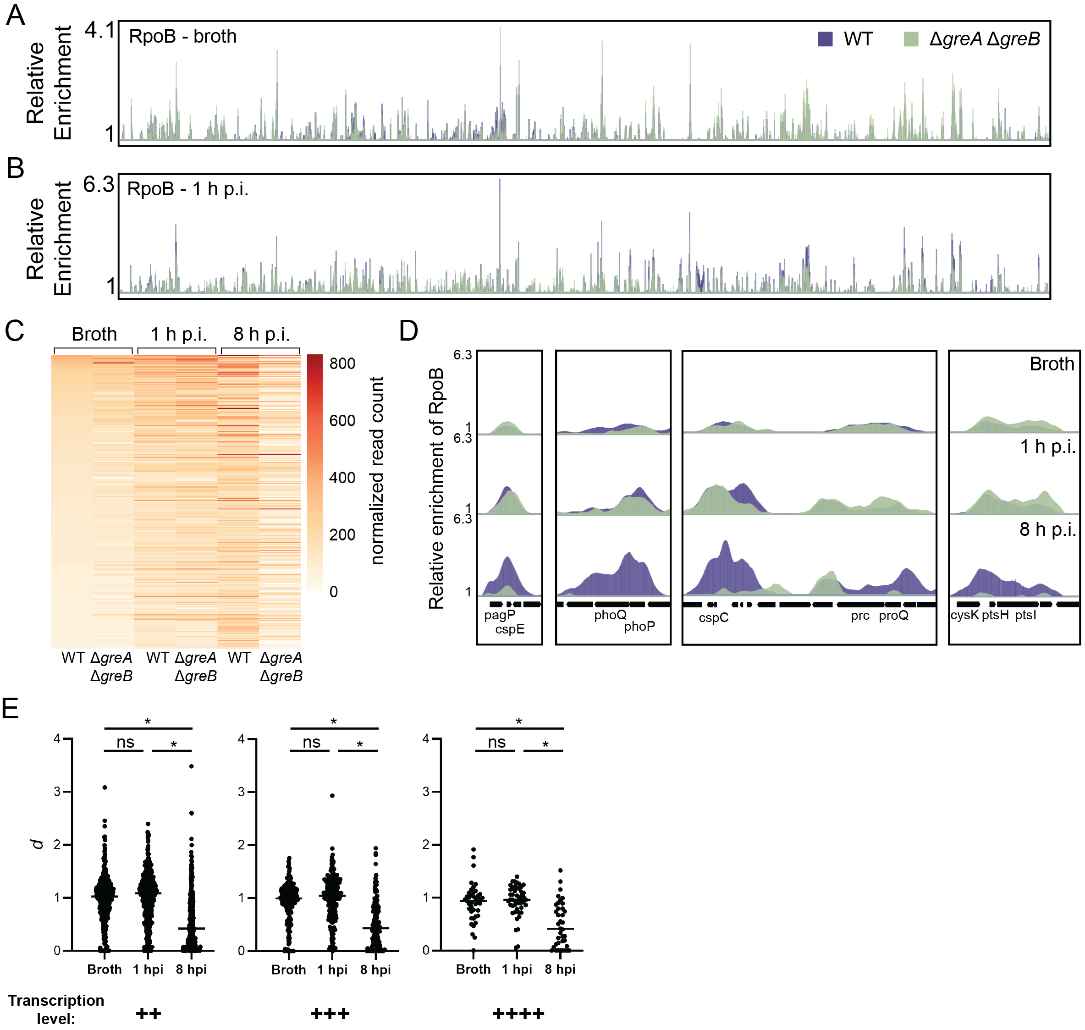


**S4 Figure**. **RNAP occupancy changes in the presence and absence of Gre factors.** Representative RNAP occupancy profile for wild-type (WT) cells or cells lacking Gre factors (Δ*greA* Δ*greB*) in cells (A) grown in broth and (B) at 1 h post infection (h p.i.) as determined by ChIP-seq of RpoB. Relative enrichment is defined as the ratio of IP and input read counts normalized to total reads. (C) RNAP occupancy changes visualized as a heat map, where every horizontal line represents the normalized read count for the same gene across each condition. Only top-transcribed genes that arise in all three conditions are plotted (283 genes). Each value represented is the average of three independent replicates. (D) RNAP occupancy profile for representative genes that are classified as top transcribed in all three conditions. The genes shown represent some of the most pronounced and pertinent differences in RNAP occupancy between conditions (see S4 Table for genes with less pronounced differences). Relative enrichment is defined as in (A) and (B). (E) Quantification of *d* ratios calculated for each gene falling within each transcription level (see S4 Table). The number of genes belonging to each condition within each transcription level is as follows: ++, 354 (broth), 412 (1 h p.i.), 405 (8 h p.i.); +++, 160 (broth), 164 (1 h p.i.), 147 (8 h p.i.); +++, 37 (broth), 42 (1 h p.i.), 38 (8 h p.i.). Each value represents the average of three independent replicates. **p*<0.0001, one-way ANOVA.


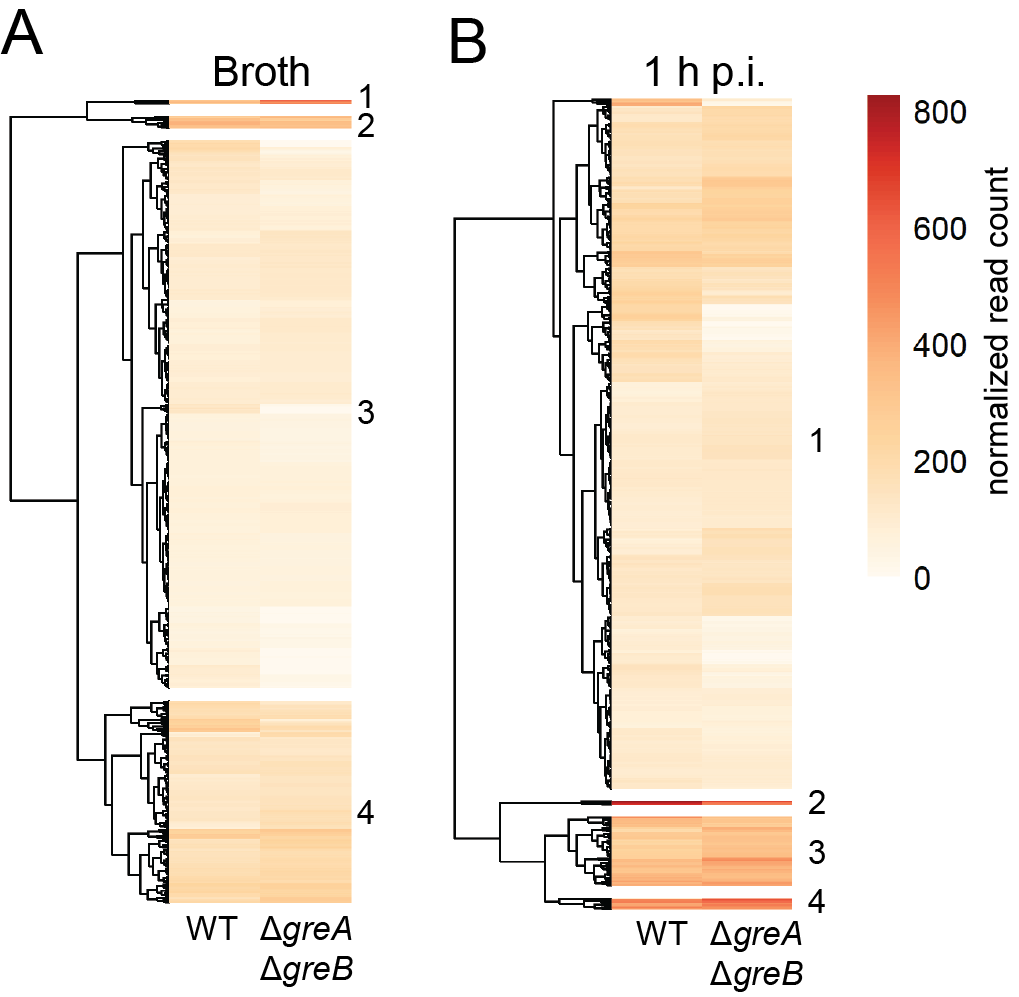


**S5 Figure. RNAP occupancy differences in broth and 1 h p.i.** RNAP occupancy changes in cells grown in broth (A) and at 1 h post infection (h p.i.) (B) in the absence of Gre factors visualized as a heat map. Each value represents the average of three independent replicates. Hierarchical clustering (numbered 1-4) was performed using the pheatmap function in RStudio (see Table S4).


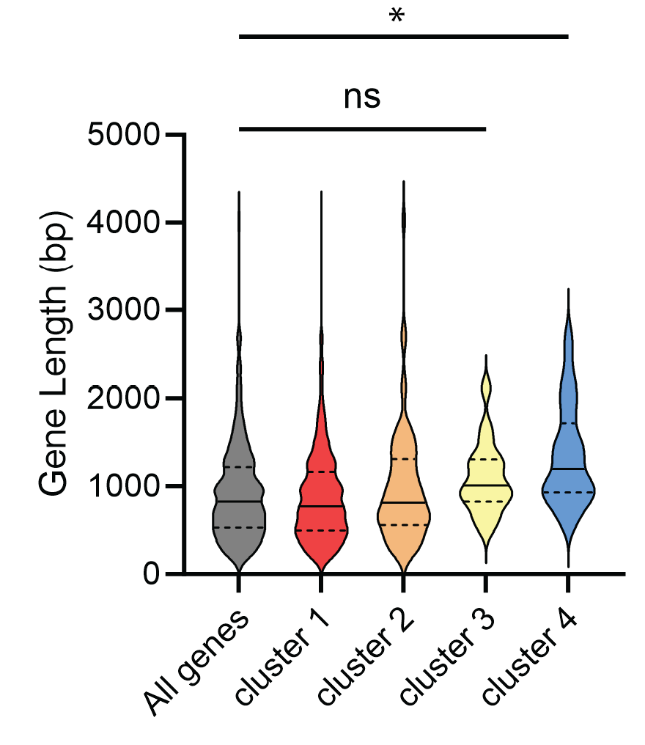


**S6 Figure. Longer genes experience more prevalent backtracking.** All top transcribed genes at 8 h post infection were sorted by hierarchical clustering using the pheatmap function in RStudio (590 genes total). Gene length was calculated for all genes in each cluster and plotted, where the median is shown by a solid line and quartiles by dashed lines. **p*<0.001, ns: not significant, one-way ANOVA.

**
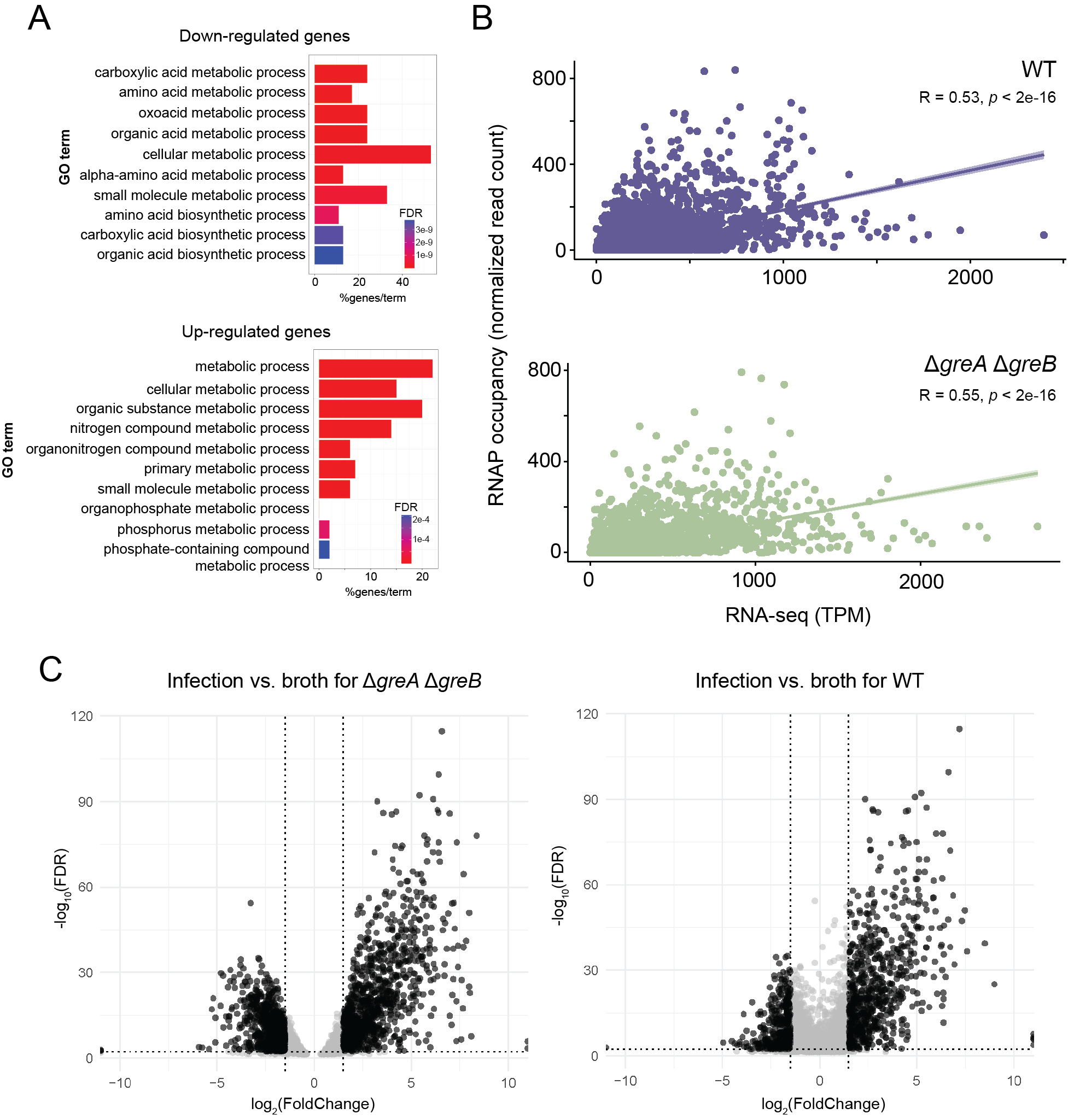
**

**S7 Figure. Summary of RNA-seq results.** (A) Top ten gene ontology (GO) terms overrepresented in downregulated genes and underrepresented in upregulated genes. %genes/term is defined as the number of genes belonging to the GO term found in the dataset (see S6 Table) versus the total number of genes of the GO term. FDR = false discovery rate (*p* value corrected for multiple testing). (B) Linear regression analysis comparing gene expression (as determined by RNA-seq data) and RNAP occupancy (as determined by RpoB PIC-seq) during infection (at 8 h post infection). Transcripts per million (TPM) and normalized read count for each gene were calculated as explained in the methods. Pearson’s correlation coefficients (R) were determined to be 0.53 and 0.55 for WT and Δ*greA* Δ*greB*, respectively. Shaded area around each line indicates 95% confidence interval. Each dot represents the mean value from two independent replicates. (C) Volcano plot of the distribution of differentially expressed genes in infection versus broth for Δ*greA* Δ*greB* cells (left) or for WT cells (right) upon DEseq2 analysis. Each dot represents the mean value of one gene from two (infection) or three (broth) independent replicates. Black color indicates significant results (FDR < 0.05, |log_2_FC| > 1.5).

**
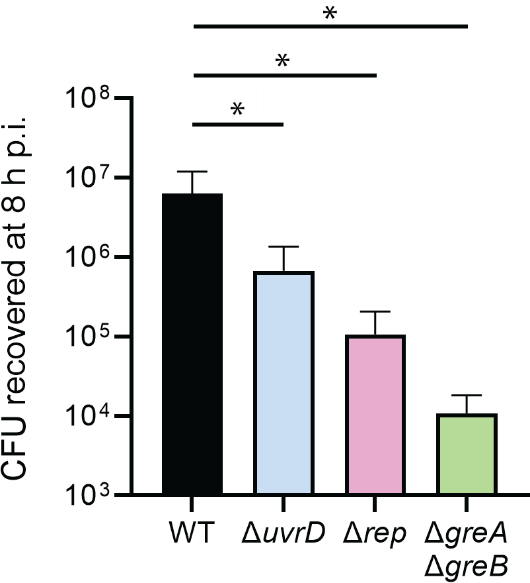
**

**S8 Figure. Cells lacking key conflict resolution factors exhibit significant growth defects during infection.** HeLa cells were infected with *S.* Typhimurium cells lacking the indicated genes. Bacteria were harvested at 8 h post infection (p.i.) and plated for CFU enumeration. **p*<0.05, one-way ANOVA.

| **Locus** | **Fold Enrichment** | **Localization** | **Function** | **Reference** |
| --- | --- | --- | --- | --- |
| *mig-14* | 2.82 | Chromosome | antimicrobial peptide resistance | ^1^ |
| *virK* | 2.79 | Chromosome | antimicrobial peptide resistance | ^2^ |
| *sifB* | 2.70 | Chromosome | putatively contributes to the formation of the intracellular replication niche | ^3^ |
| *sitD* | 2.62 | SPI-1 | resistance to nitrosative stress | ^4^ |
| *SL1344_1344* | 2.57 | Chromosome | putative component of the type III secretion system (T3SS-2) apparatus | ^5^ |
| *ssaK* | 2.57 | SPI-2 | T3SS-2 apparatus | ^6^ |
| *sitC* | 2.53 | SPI-1 | resistance to nitrosative stress | ^4^ |
| *pdgL* | 2.53 | Chromosome | putative role in cell wall/membrane biogenesis | ^7^ |
| *SL1344_2902* | 2.49 | Chromosome | hypothetical protein |  |
| *rplC* | 2.49 | Chromosome | ribosomal protein L3 |  |
| *ssaH* | 2.46 | SPI-2 | putative role in T3SS-2 apparatus | ^8^ |
| *ssaI* | 2.46 | SPI-2 | putative role in T3SS-2 apparatus | ^8^ |
| *ssaJ* | 2.46 | SPI-2 | T3SS-2 apparatus | ^6^ |
| *pipB2* | 2.41 | Chromosome | kinesin accumulation in the SCV | ^9^ |
| *ssaE* | 2.40 | SPI-2 | chaperone | ^10^ |
| *SL1344_1530A* | 2.37 | Chromosome | hypothetical protein |  |
| *ugtL* | 2.37 | Chromosome | PhoP activation | ^11^ |
| *sitA* | 2.36 | SPI-1 | resistance to nitrosative stress | ^4^ |
| *sitB* | 2.36 | SPI-1 | resistance to nitrosative stress | ^4^ |
| *sseB* | 2.34 | SPI-2 | T3SS-2 translocon | ^12^ |
| *sscA* | 2.34 | SPI-2 | chaperone | ^13^ |
| *sseD* | 2.33 | SPI-2 | T3SS-2 translocon | ^12^ |
| *sseK/sseK3* | 2.27 | Chromosome | promote intracellular survival | ^14^ |
| *sseC* | 2.20 | SPI-2 | T3SS-2 translocon | ^12^ |
| *ssaL* | 2.19 | SPI-2 | T3SS-2 apparatus | ^6^ |
| *mgtC* | 2.18 | SPI-3 | maintenance of ATP homeostasis | ^15^ |
| *ssaR* | 2.18 | SPI-2 | T3SS-2 apparatus | ^6^ |
| *sseG* | 2.17 | SPI-2 | SIF biogenesis and SCV maintenance | ^16^ |
| *iscS* | 2.15 | Chromosome | tRNA synthesis | ^17^ |
| *prgJ* | 2.15 | SPI-1 | T3SS-1 | ^18^ |
| *prgI* | 2.15 | SPI-1 | T3SS-1 | ^18^ |
| *ssaQ* | 2.10 | SPI-2 | T3SS-2 apparatus | ^19^ |

**S1 Table. RNAP is significantly enriched at virulence genes during infection.** Loci where RpoB was significantly enriched at least two-fold in *S.* Typhimurium 8 h post infection as determined by PIC-seq. Each value represents the average fold enrichment (‘signalValue’ output of macs2) for at least two independent replicates.

|  | **Fold Enrichment** | |
| --- | --- | --- |
| **Locus** | **Broth** | **Infection** |
| *yiaG* | 2.01 | 3.44 |
| *cspA* | 2.01 | 3.44 |
| *cueP* | 2.01 | 3.44 |
| *deaD* | 2.47 | 2.93 |
| *yrbN* | 2.47 | 2.93 |
| *nlpI* | 2.47 | 2.93 |
| *pnp* | 2.47 | 2.90 |
| *cspE (cspC)* | 2.23 | 2.84 |
| *yobF* | 2.23 | 2.84 |
| *SL1344_RS09185* | 2.23 | 2.82 |
| *mgrB (yobG)* | 2.23 | 2.82 |
| *yqcC* | 2.75 | 2.69 |
| *hdfR* | 2.33 | 2.50 |
| *trxA* | 2.07 | 2.35 |
| *rhoL* | 2.07 | 2.35 |
| *rho* | 2.07 | 2.35 |
| *rpsD* | 2.08 | 2.33 |
| *rpsK* | 2.08 | 2.33 |
| *rpsM* | 2.08 | 2.33 |
| *rpmJ* | 2.08 | 2.33 |
| *rplN* | 2.08 | 2.31 |
| *rplO* | 2.08 | 2.27 |
| *secY/prlA* | 2.08 | 2.26 |
| *rpoA/pez* | 2.08 | 2.18 |
| *rpsC* | 2.21 | 2.18 |
| *rplV* | 2.21 | 2.18 |
| *rplB* | 2.21 | 2.13 |
| *rpoH* | 2.07 | 2.12 |
| *rluD* | 2.37 | 2.08 |
| *pgeF* | 2.21 | 2.03 |

**S2 Table.** **RNAP is significantly enriched at some of the same genes in broth and infection**. Loci where RpoB was significantly enriched at least two-fold in S. Typhimurium in cells grown in broth and 8 h post infection, as determined by PIC-seq. Each value represents the average fold enrichment (‘signalValue’ output of macs2) for at least two independent replicates.

*Excel file*

**S3 Table. PIC-seq summary.** List of the raw read counts from featureCounts analysis for every feature, the reads normalized to total read count for both IP and input, normalized read count, and the average normalized read count across three biological replicates. See methods.

*Excel file*

**S4 Table. Top transcribed genes in cells grown in broth, 1 h p.i., and 8 h p.i.** List of the top transcribed genes as determined by k-means clustering for each condition. Values represent the average normalized read count and the ratio of the normalized read counts of three independent replicates. This table also lists the 283 top transcribed genes that arise in all three conditions.

*Excel file*

**S5 Table. RNAP occupancy changes for the top transcribed genes as categorized by hierarchical clustering.** Lists the top transcribed genes for each condition that fall into each hierarchical cluster, as determined by pheatmap function in R. Values represent the average normalized read count and the ratio of the normalized read counts of three independent replicates. Also lists the manually annotated functions of genes in clusters three and four for the 8 h post infection (p.i.) condition.

*Excel file*

**S6 Table. RNA-seq summary.** Tab 1 shows raw read counts per gene for each individual replicate for both conditions (broth and 8 hours post infection [p.i.]). An average of 588,000 reads mapped to features in the infection condition, approximately 1.5 million reads mapped to features in the broth condition. Tab 2 shows TPM calculated for every gene per individual replicate for both conditions (see methods). Tab 3 shows differential expression as determined by DEseq2 for Δ*greA* Δ*greB* cells versus WT cells at 8 h p.i. Only genes with more than ten mapped reads are included (total = 4504 genes). Tab 4 shows differential expression of genes in SPI-1 and SPI-2. Tab 5 shows differential expression analysis of only those genes where differential expression was statistically significant (FDR < 0.05, total = 1184 genes). Tab 6 shows differential expression analysis of only those genes where the differential expression (as determined by log_2_ of the fold change) differed by more than two-fold (log_2_FoldChange ≥ |1|, total = 713 genes). Tab 7 shows differential expression analysis of genes from Tab 6 that are upregulated (344 genes). Tab 8 shows differential expression analysis of genes from Tab 7 that are downregulated (369 genes).

**S7 Table. Strains, plasmids, and primers used in this study.**

| **Strain** | **Genotype/Features** | **Identifier** | **Reference/Source** |
| --- | --- | --- | --- |
| SL1344 | Wild-type *S.* Typhimurium strain SL1344 | HM4315 | This study |
| Δ*greB* | *greB::Cat* | HM4525 | This study |
| Δ*greA* | *greA::Kan* | HM4527 | This study |
| Δ*greA* Δ*greB* | *greA::Kan greB::Cat* | HM4529 | This study |
| T-SACK | W3110 araD<>tetA-sacB amp fliC<>cat argG::Tn5 | HM4177 | ^20^ |
|  |  |  |  |
| **Plasmid** | **Features** | **Reference/Source** | |
| pSIM27 | For expression of Red recombinase system | Gift from Dr. Don Court | |
|  |  |  | |
| **Primers** | **Sequence** | **Description** | |
| HM6042 | CATTGCCCCCTACAGGAATGTTCAAGAGG-TATAACAAATGTATGGACAGCAAGCGAACCG | For recombineering greA with the Kanamycin cassette from T-SACK | |
| HM6043 | TTTACAATACACCAACAATTTGCGTATTGAG-TACTGCTTATCAGAAGAACTCGTCAAGAAG | For recombineering greA with the Kanamycin cassette from T-SACK | |
| HM6040 | GTGTGCGCAATATCGACAGCAAAGGTAAAT-CAACGAGATGTGTGACGGAAGATCACTTCG | For recombineering greB with the Chloramphenicol cassette from T-SACK | |
| HM6041 | TGCCAGCCATCAGCGGGGGCTTAGGATTC-TTCTTGTCTTAACCAGCAATAGACATAAGCG | For recombineering greB with the Chloramphenicol cassette from T-SACK | |
| HM5726 | TCGACCGGTGATAATTCGCT | *S.* Typhimurium *eutN* qPCR | |
| HM5727 | CGCTATCGACAGTATCGGGG | *S.* Typhimurium *eutN* qPCR | |
| HM6792 | GCCCGAGTTGGATCGTCTTC | *S.* Typhimurium *ssaK* qPCR | |
| HM6793 | CTGCCGTTTTTGCCTGTCAT | *S.* Typhimurium *ssaK* qPCR | |
| HM6788 | CGGTTGCTATGCCAATCTGC | *S.* Typhimurium *gstA* qPCR | |
| HM6789 | CGGTTATGTCGCCAACCTGA | *S.* Typhimurium *gstA* qPCR | |
| HM6784 | AATCCAGCGGCCCTATTTGT | *S.* Typhimurium *virK* qPCR | |
| HM6785 | TCCGGAAAGGACTGAACGAA | *S.* Typhimurium *virK* qPCR | |
| HM7065 | CGAACGCCGTGAGTTTGATG | *S.* Typhimurium *phoP* qPCR | |
| HM7066 | TAATGCGCCGTAATAGCGGT | *S.* Typhimurium *phoP* qPCR | |

**References**

1. Brodsky, I. E., Ernst, R. K., Miller, S. I. & Falkow, S. mig-14 Is a Salmonella Gene That Plays a Role in Bacterial Resistance to Antimicrobial Peptides. *J. Bacteriol.* **184**, 3203–3213 (2002).

2. Detweiler, C. S., Monack, D. M., Brodsky, I. E., Mathew, H. & Falkow, S. virK, somA and rcsC are important for systemic Salmonella enterica serovar Typhimurium infection and cationic peptide resistance. *Mol. Microbiol.* **48**, 385–400 (2003).

3. Freeman, J. A., Ohl, M. E. & Miller, S. I. The Salmonella enterica Serovar Typhimurium Translocated Effectors SseJ and SifB Are Targeted to the Salmonella-Containing Vacuole. *Infect. Immun.* **71**, 418–427 (2003).

4. Yousuf, S. *et al.* Manganese import protects Salmonella enterica serovar Typhimurium against nitrosative stress†. *Metallomics* **12**, 1791–1801 (2020).

5. Mazé, A., Glatter, T. & Bumann, D. The Central Metabolism Regulator EIIA Glc Switches Salmonella from Growth Arrest to Acute Virulence through Activation of Virulence Factor Secretion. *Cell Rep.* **7**, 1426–1433 (2014).

6. Hensel, M. *et al.* Functional analysis of ssaJ and the ssaK/U operon, 13 genes encoding components of the type III secretion apparatus of Salmonella Pathogenicity Island 2. *Mol. Microbiol.* **24**, 155–167 (1997).

7. Yoon, H., McDermott, J. E., Porwollik, S., McClelland, M. & Heffron, F. Coordinated Regulation of Virulence during Systemic Infection of Salmonella enterica Serovar Typhimurium. *PLoS Pathog.* **5**, e1000306 (2009).

8. Chakravortty, D., Rohde, M., Jäger, L., Deiwick, J. & Hensel, M. Formation of a novel surface structure encoded by Salmonella Pathogenicity Island 2. *EMBO J.* **24**, 2043–2052 (2005).

9. Fàbrega, A. & Vila, J. Salmonella enterica serovar Typhimurium skills to succeed in the host: virulence and regulation. Clin. Microbiol. Rev. 26, 308–341 (2013).

10. Miki, T., Shibagaki, Y., Danbara, H. & Okada, N. Functional Characterization of SsaE, a Novel Chaperone Protein of the Type III Secretion System Encoded by Salmonella Pathogenicity Island 2. *J. Bacteriol.* **191**, 6843–6854 (2009).

11. Choi, J., Salvail, H. & Groisman, E. A. RNA chaperone activates Salmonella virulence program during infection. *Nucleic Acids Res.* **49**, 11614–11628 (2021).

12. Nikolaus, T. *et al.* SseBCD proteins are secreted by the type III secretion system of Salmonella pathogenicity island 2 and function as a translocon. *J. Bacteriol.* **183**, 6036–6045 (2001).

13. Zurawski, D. V. & Stein, M. A. The SPI2-encoded SseA chaperone has discrete domains required for SseB stabilization and export, and binds within the C-terminus of SseB and SseD. *Microbiology* **150**, 2055–2068 (2004).

14. Günster, R. A., Matthews, S. A., Holden, D. W. & Thurston, T. L. M. SseK1 and SseK3 Type III Secretion System Effectors Inhibit NF-κB Signaling and Necroptotic Cell Death in Salmonella-Infected Macrophages. *Infect. Immun.* **85**, e00010-17 (2017).

15. Lee, J.-W. & Lee, E.-J. Regulation and function of the Salmonella MgtC virulence protein. *J. Microbiol. Seoul Korea* **53**, 667–672 (2015).

16. Knuff-Janzen, K., Tupin, A., Yurist-Doutsch, S., Rowland, J. L. & Finlay, B. B. Multiple Salmonella-pathogenicity island 2 effectors are required to facilitate bacterial establishment of its intracellular niche and virulence. *PLoS ONE* **15**, e0235020 (2020).

17. Lundgren, H. K. & Björk, G. R. Structural alterations of the cysteine desulfurase IscS of Salmonella enterica serovar Typhimurium reveal substrate specificity of IscS in tRNA thiolation. *J. Bacteriol.* **188**, 3052–3062 (2006).

18. Lou, L., Zhang, P., Piao, R. & Wang, Y. Salmonella Pathogenicity Island 1 (SPI-1) and Its Complex Regulatory Network. *Front. Cell. Infect. Microbiol.* **9**, (2019).

19. Yu, X.-J., Liu, M., Matthews, S. & Holden, D. W. Tandem translation generates a chaperone for the Salmonella type III secretion system protein SsaQ. *J. Biol. Chem.* **286**, 36098–36107 (2011).

20. Li, X., Thomason, L. C., Sawitzke, J. A., Costantino, N. & Court, D. L. Positive and negative selection using the tetA-sacB cassette: recombineering and P1 transduction in Escherichia coli. *Nucleic Acids Res.* **41**, e204 (2013).
